# Posicionamento sobre Indicações e Reintrodução dos Métodos de Imagem Cardiovascular de Forma Segura no Cenário da COVID-19 – 2021

**DOI:** 10.36660/abc.20210133

**Published:** 2021-03-03

**Authors:** Adenalva Lima de Souza Beck, Silvio Henrique Barberato, André Luiz Cerqueira de Almeida, Claudia R. Pinheiro de Castro Grau, Marly Maria Uellendahl Lopes, Ronaldo de Souza Leão Lima, Rodrigo Júlio Cerci, Ana Cristina Lopes Albricker, Fanilda Souto Barros, Alessandra Joslin Oliveira, Edgar Bezerra de Lira, Marcelo Haertel Miglioranza, Marcelo Luiz Campos Vieira, José Luiz Barros Pena, Tânia Mara Varejão Strabelli, David Costa de Souza Le Bihan, Jeane Mike Tsutsui, Carlos Eduardo Rochitte

**Affiliations:** 1 Universitária de Cardiologia Instituto de Cardiologia do Distrito Federal BrasíliaDF Brasil Instituto de Cardiologia do Distrito Federal - Fundação Universitária de Cardiologia, Brasília, DF – Brasil; 2 Hospital Sírio-Libanês BrasíliaDF Brasil Hospital Sírio-Libanês, Brasília, DF – Brasil; 3 CardioEco - Centro de Diagnóstico CuritibaPR Brasil CardioEco - Centro de Diagnóstico Cardiovascular, Curitiba, PR – Brasil; 4 Quanta Diagnóstico e Terapia CuritibaPR Brasil Quanta Diagnóstico e Terapia, Curitiba, PR – Brasil; 5 Santa Casa de Misericórdia de Feira de Santana Feira de SantanaBA Brasil Santa Casa de Misericórdia de Feira de Santana, Feira de Santana, BA – Brasil; 6 Hospital das Clínicas da Faculdade de Medicina da Universidade de São Paulo Instituto do Coração (InCor) São PauloSP Brasil Instituto do Coração (InCor) do Hospital das Clínicas da Faculdade de Medicina da Universidade de São Paulo (HCFMUSP), São Paulo, SP - Brasil; 7 Grupo Fleury São PauloSP Brasil Grupo Fleury, São Paulo, SP – Brasil; 8 Universidade Estadual Paulista São PauloSP Brasil Universidade Estadual Paulista (Unesp), São Paulo, SP – Brasil; 9 Diagnósticos da América São PauloSP Brasil Diagnósticos da América SA (Dasa), São Paulo, SP – Brasil; 10 Universidade Federal do Rio de Janeiro Rio de JaneiroRJ Brasil Universidade Federal do Rio de Janeiro (UFRJ), Rio de Janeiro, RJ – Brasil; 11 Instituto Mineiro de Ultrassonografia Belo HorizonteMG Brasil Instituto Mineiro de Ultrassonografia (IMEDE), Belo Horizonte, MG – Brasil; 12 VitóriaES Brasil Vascular Vitória S/C LTDA., Vitória, ES – Brasil; 13 Hospital Israelita Albert Einstein São PauloSP Brasil Hospital Israelita Albert Einstein, São Paulo, SP – Brasil; 14 Hospital Mãe de Deus Porto AlegreRS Brasil Prevencor – Hospital Mãe de Deus, Porto Alegre, RS – Brasil; 15 Fundação Universitária de Cardiologia Instituto de Cardiologia do Rio Grande do Sul Porto AlegreRS Brasil Instituto de Cardiologia do Rio Grande do Sul – Fundação Universitária de Cardiologia, Porto Alegre, RS – Brasil; 16 Faculdade Ciências Médicas de Minas Gerais Belo HorizonteMG Brasil Faculdade Ciências Médicas de Minas Gerais, Belo Horizonte, MG – Brasil; 17 Hospital Felício Rocho Belo HorizonteMG Brasil Hospital Felício Rocho, Belo Horizonte, MG – Brasil; 18 Instituto Dante Pazzanese de Cardiologia São PauloSP Brasil Instituto Dante Pazzanese de Cardiologia, São Paulo, SP – Brasil; 19 Hospital do Coração São PauloSP Brasil Hospital do Coração (HCor), São Paulo, SP – Brasil; 20 Hospital Pró-Cardíaco Rio de JaneiroRJ Brasil Hospital Pró-Cardíaco, Rio de Janeiro, RJ – Brasil

**Table:** Declaração de potencial conflito de interesses dos autores/colaboradores do Posicionamento sobre Indicações e Reintrodução dos Métodos de Imagem Cardiovascular de Forma Segura no Cenário da COVID-19 – 2021

Se nos últimos 3 anos o autor/colaborador do posicionamento:							
Nome do integrante do posicionamento	Participou de estudos clínicos e/ou experimentais subvencionados pela indústria farmacêutica ou de equipamentos relacionados ao posicionamento em questão	Foi palestrante em eventos ou atividades patrocinadas pela indústria relacionados ao posicionamento em questão	Foi (é) membro do conselho consultivo ou diretivo da indústria farmacêutica ou de equipamentos	Participou de comitês normativos de estudos científicos patrocinados pela indústria	Recebeu auxílio pessoal ou institucional da indústria	Elaborou textos científicos em periódicos patrocinados pela indústria	Tem ações da indústria
Adenalva Lima de Souza Beck	Não	Não	Não	Não	Não	Não	Não
Alessandra Joslin Oliveira	Não	Não	Não	Não	Não	Não	Não
Ana Cristina Lopes Albricker	Não	Não	Não	Não	Não	Não	Não
André Luiz Cerqueira de Almeida	Não	Não	Não	Não	Não	Não	Não
Carlos Eduardo Rochitte	Não	Não	Não	Não	Não	Não	Não
Claudia R. Pinheiro de Castro Grau	Não	Não	Não	Não	Não	Não	Não
David Costa de Souza Le Bihan	Não	Não	Não	Não	Não	Não	Não
Edgar Bezerra de Lira Filho	Não	Não	Não	Não	Não	Não	Não
Fanilda Souto Barros	Não	Não	Não	Não	Não	Não	Não
Jeane Mike Tsutsui	Não	Não	Não	Não	Não	Não	Não
José Luiz Barros Pena	Não	Não	Não	Não	Não	Não	Não
Marcelo Haertel Miglioranza	Não	Não	Não	Não	Não	Não	Não
Marcelo Luiz Campos Vieira	Não	Não	Não	Não	Não	Não	Não
Marly Maria Uellendahl Lopes	Não	Não	Não	Não	Não	Não	Não
Rodrigo Júlio Cerci	Não	Não	Não	Não	Não	Não	Não
Ronaldo de Souza Leão Lima	Não	Não	Não	Não	Não	Não	Não
Silvio Henrique Barberato	Não	Não	Não	Não	Não	Não	Não
Tânia Mara Varejão Strabelli	Não	Não	Não	Não	Não	Não	Não

## Carta de Apresentação

O objetivo deste posicionamento é informar clínicos, cardiologistas e imaginologistas sobre procedimentos, fluxos e protocolos recomendados para o período da pandemia do SARS-CoV-2 (COVID-19), visando à proteção mais eficiente de profissionais de saúde e pacientes. As recomendações são baseadas na melhor evidência científica disponível no momento e no consenso de especialistas com grande experiência na área. Desde o primeiro caso de COVID-19 no Brasil, passamos por inúmeras mudanças nas recomendações e grande debate científico sobre algumas delas nas diversas fontes de conhecimento científico. Isso se deve ao conhecimento incompleto da doença COVID-19, incluindo processos fisiopatológicos e de transmissão do SARS-CoV-2. Outro motivo da variabilidade das recomendações está relacionado à fase epidemiológica em que se encontra a pandemia em determinada região do país. É notório que, em um país continental como o Brasil, a pandemia poderá estar em fases distintas de transmissão em um momento específico, requerendo, portanto, medidas particularizadas para cada região e fase da pandemia.

No início da pandemia, em fase de grande atenção e desconhecimento do que o futuro nos reservaria, o Departamento de Imagem Cardiovascular (DIC) da Sociedade Brasileira de Cardiologia (SBC) publicou um posicionamento em versão abreviada e essencial para aquele momento da pandemia, fornecendo, de maneira rápida e prática, orientações fundamentais de segurança nos procedimentos de imagem cardiovascular não invasiva. Esse documento foi absolutamente essencial naquele instante para preservar ao máximo nossos profissionais da área. Após a publicação de documento sumário na fase inicial da pandemia, o DIC/SBC e seus especialistas julgaram oportuna a atualização daquele posicionamento (agora na forma de publicação científica oficial), que incluiu não somente uma visão muito mais ampla dos procedimentos à luz do conhecimentos adquiridos desde então, mas também uma orientação customizada à fase epidemiológica da pandemia e que poderá ser útil aos profissionais de imagem não invasiva cardiovascular para os possíveis meses e anos à frente, em que provavelmente conviveremos com a COVID-19.

## 1. Introdução

Diante da pandemia da COVID-19 e sua alta transmissibilidade, fez-se necessária uma urgente reorganização dos serviços de imagem cardiovascular para minimizar a exposição ao novo coronavírus (SARS-CoV-2) e assegurar a proteção de pacientes, médicos e equipe de trabalho, sem, entretanto, comprometer a assistência ao paciente. Foi recomendado postergar exames eletivos ambulatoriais (quando considerados não essenciais), a fim de minimizar exposição e risco de transmissão cruzada, além de racionalizar o uso de equipamentos de proteção individual (EPI).^1^^–^^3^ A utilização de EPI passou a ser imprescindível para todos os que circulam na área de trabalho, incluindo recepcionistas, técnicos de enfermagem, enfermeiros, tecnólogos, biomédicos e médicos. O uso de máscaras pelos pacientes passou a ser obrigatório. Os especialistas passaram a discutir conjuntamente com seus médicos solicitantes a real necessidade da urgência dos exames e qual a modalidade de imagem cardiovascular mais adequada naquela situação clínica específica. Por outro lado, sabe-se que (a) o exame de imagem cardiovascular é muitas vezes necessário para prevenção primária, manejo clínico e diagnóstico diferencial em várias situações, e postergar cronicamente a sua realização pode ser prejudicial; (b) a COVID-19 pode gerar manifestações cardiovasculares graves, especialmente nos indivíduos mais vulneráveis, tais como idosos, imunossuprimidos ou com doença cardiovascular prévia e/ou fatores de risco cardiovasculares (hipertensão, diabetes, obesidade);^4^^–^^6^ (c) quando os pulmões são gravemente acometidos pela COVID-19, pode haver maior impacto na função cardíaca, especialmente no ventrículo direito. No momento em que os registros de casos da doença apresentarem relativo decaimento da curva, os serviços poderão progressivamente ampliar os seus horários de atendimento de acordo com a tendência da pandemia e a orientação das autoridades públicas de cada local. Contudo, a reintrodução dos exames de imagem cardiovascular deve seguir vários protocolos de segurança, discutidos a seguir.

## 2. Protocolos de Segurança para Reintrodução de Exames de Imagem Cardiovascular na Era COVID-19

O agendamento ambulatorial deve ser progressivo, considerando as indicações de uso apropriado,^2^^,^^7^ a prioridade da indicação, o risco de o indivíduo ter COVID-19 e a fase da pandemia. Nos locais que estão no pico da pandemia, deve-se priorizar a realização daqueles exames considerados essenciais (alta prioridade), ou seja, onde se espera que o resultado traga benefício clínico ou mudança de conduta.^1^^,^^8^ Indicações consideradas de média prioridade são aquelas em que a realização dos exames, apesar de eletivos, ou em pacientes assintomáticos, pode auxiliar na implementação de medidas de prevenção primária ou secundária, ajuste de medicações em uso ou mudança de conduta a médio prazo, com potencial de obter impacto no desfecho clínico. Indicações consideradas de baixa prioridade são aquelas em que o agendamento do exame pode ser postergado para após o pico da pandemia e reintroduzido gradualmente com a redução do número de casos.

A realização de exames de imagem em pacientes com COVID-19, principalmente se forem ambulatoriais, deve ser postergada, sempre que possível, para quando forem obtidos os critérios de cura. No momento em que escrevemos esse posicionamento, o Centers for Disease Control and Prevention (CDC) considera que existem dois critérios para liberar pacientes do isolamento após COVID-19: a “estratégia baseada em sintomas”, que considera liberados os pacientes 10 dias após o início dos sintomas e pelo menos 72 horas assintomáticos, e a estratégia baseada em tempo”, que considera liberados os pacientes 10 dias após do exame positivo (RT-PCR SARS-Cov-2).^9^ Esses critérios podem ser ajustados conforme as orientações da comissão de infecção local. Se o exame for considerado essencial, deve ser direcionado para a questão clínica (focado), porém completo o suficiente para evitar a repetição, e seguir todas as recomendações quanto às medidas de proteção. Além disso, testes redundantes ou raramente apropriados podem gerar impacto financeiro adicional ao causado pela pandemia. Essas recomendações sobre indicações e priorização são válidas enquanto perdurar a pandemia e estão resumidas na Tabela 1.

**Tabela 1:** Priorização de indicações para reagendamento de exames de imagem cardiovascular de acordo com a fase da pandemia e o risco de COVID-19

Nível de prioridade	Racional para priorização
Alta prioridade (Considere realizar o exame nas próximas horas ou em até 2 a 4 semanas)	Sintomas cardiovasculares agudos ou com piora recente Avaliação antes de terapia clínica urgente Planejamento de intervenção cardiovascular urgente Monitoramento da segurança de terapia clínica Controle após terapia cirúrgica ou invasiva recente **Realizar independentemente da fase da pandemia**
Moderada prioridade	Monitoramento da progressão de doença crônica miocárdica ou valvar grave assintomática Terapia clínica que requer monitoramento Monitoramento do resultado de terapia Avaliação inicial de novo sopro inexplicado, mesmo que assintomático **Postergar para a fase de desaceleração da pandemia e preferencialmente para pacientes com baixo risco de COVID-19**
Baixa prioridade	Avaliação de rotina de doença crônica em indivíduos não elegíveis para terapia clínica, cirúrgica ou invasiva **Postergar para a fase de controle da pandemia e em pacientes com baixo risco de COVID-19**

*Tabela adaptada das recomendações da Sociedade Americana de Ecocardiografia*.^*10*^

Definir “em quem”, “quando” e “como” o exame de imagem cardiovascular deve ser utilizado é fundamental para diminuir os riscos de contaminação para o paciente e profissionais de saúde e, ao mesmo tempo, garantir assistência de alta qualidade. Esses cuidados são descritos a seguir.

### 2.1. Infraestrutura e Políticas de Segurança

- Questionário de triagem para definir o risco de COVID-19 deve ser aplicado no momento do agendamento, na confirmação e no dia da realização do exame (sintomas respiratórios e contato com caso confirmado de COVID-19). Na admissão, realizar também a checagem de temperatura. Mesmo protocolo de triagem deve ser aplicado diariamente aos profissionais.
- Instruções devem ser dadas no momento do agendamento quanto a medidas de distanciamento social, uso de máscaras e higiene das mãos, e reforçadas no momento da admissão.
- Implantação de estrutura de telemedicina.
- Alertas visuais quanto às medidas de proteção devem estar colocados em salas de espera e em locais estratégicos (em mais de um idioma).
- Higienizadores com álcool em gel amplamente disponíveis.
- Pacientes devem ser orientados a chegar pontualmente ou aguardar em seus carros até serem convocados, e é necessário limitar o número de acompanhantes.
- Bloqueio ou redução de assentos na sala de espera, a fim de manter o distanciamento social.
- Barreiras de acrílico ou cones de distanciamento entre paciente e equipe de atendimento.
- Aumentar o tempo de agendamento entre os exames para evitar aglomerações e facilitar a higienização. Deve-se considerar abertura de horários extracomerciais ou em fins de semana.
- Protocolos de higienização de equipamentos e superfícies a cada exame devem ser seguidos de acordo com políticas locais de controle de infecção e tipo de exame.
- Priorizar comunicados aos pacientes ou operações financeiras por meios digitais.
- É recomendado haver dois fluxos de atendimento: um para os pacientes com suspeita ou presença de COVID-19 e outro para os pacientes sem a doença. Deve-se dispor de salas, equipamentos dedicados e áreas de circulação separadas para pacientes com COVID-19.
- Idealmente dispor de salas bem ventiladas, com pressão negativa, para realização de procedimentos que gerem emissão de aerossóis (ecocardiografia transesofágica [ETE], exames sob estresse com exercício).
- Monitorar e adequar continuamente os estoques de EPI.
- Realizar descarte de material contaminado de acordo com as politicas dos órgãos de vigilância sanitária.

### 2.2. Priorização das Indicações e Escolha do Exame de Imagem Cardiovascular

- Definir a prioridade da realização do exame (de acordo com a fase da pandemia em que a região se encontra e o risco de COVID-19 [Tabela 1]).
- Escolher o melhor teste que ofereça informação essencial para a condição clínica.
- Considerar substituir um teste por outro com acurácia similar, porém com menor risco de exposição à COVID-19.
- Evitar realização de múltiplos testes ou repetição inapropriada do mesmo exame.

### 2.3. Proteção dos Profissionais

**Figura 1:**
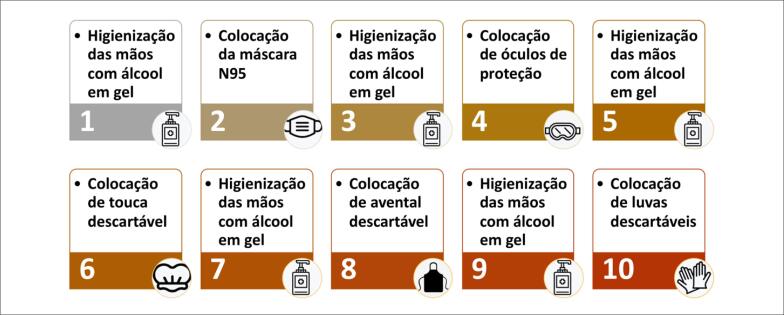
Fluxo de paramentação com equipamentos de proteção individual.

- Praticar higiene frequente das mãos e uso constante de máscaras.
- Fazer uso apropriado de EPI de acordo com o nível de proteção necessário (para gotículas ou para aerossóis). A Tabela 2 resume o uso dos EPI de acordo com o nível de proteção requerido para cada tipo de exame, risco de COVID-19 e local da realização.
- Receber treinamento institucional frequente na colocação, retirada, tempo de uso e acondicionamento de EPI, bem como higienização das mãos. Há evidências de que a maior chance de infecção do profissional ocorre no momento da remoção inadequada dos EPI. O fluxo e as orientações para colocação e retirada dos EPI estão demonstrados nas Figuras 1 e 2.
- Limitar o número de profissionais na sala de exame e na sala de laudos.
- Para exames com contato próximo entre profissional e examinador, como ecocardiografia e ultrassonografia vascular, considerar o uso de barreira de acrílico ou de plástico entre paciente e profissional.
- Limitar o tempo de exame optando por protocolos mais objetivos ou focados. No caso de pacientes internados com suspeita ou doença ativa, os exames de ultrassom/ecocardiografia deverão ser realizados à beira do leito, sem monitoramento eletrocardiográfico, e deve-se obter as imagens e realizar as medidas após saída do quarto/sala de exame e higienização do aparelho.
- Concentrar a realização de pacientes internados com suspeita ou confirmação de COVID-19, se possível dentro de um mesmo período para minimizar a exposição e racionalizar o uso de EPI.

**Tabela 2:** Uso de equipamentos de proteção individual durante a realização de exames de imagem cardiovascular na era COVID-19 de acordo com o risco de exposição

	ETT, TCC/RMC, estresse farmacológico (Eco/SPECT/PET/RMC)	ETE, estresse com exercício (Eco/SPECT/cintilografia)
Baixo risco de COVID-19	Nível de proteção padrão Higienização obrigatória das mãos Máscara cirúrgica	Nível de proteção para aerossóis:** Higienização obrigatória das mãos Máscara N-95/PFF2* Avental de isolamento (impermeável, de preferência) Luvas de procedimento Touca cirúrgica Protetor facial (*face shield*) ou óculos de proteção, se não houver protetor facial disponível Em pacientes portadores de COVID-19, realizar idealmente em sala com pressão negativa Tentar substituir por método alternativo
Suspeitos e portadores de COVID-19	Nível de proteção para gotículas Higienização obrigatória das mãos Máscara N-95/PFF2* Avental de isolamento Gorros Luvas de procedimento Óculos de proteção	

* *Na indisponibilidade total de máscara N-95 ou similares, utilizar máscara cirúrgica de uso único acompanhado de protetor facial (face shield)*.

** *Aplicar também a qualquer exame realizado em paciente internados em unidade de terapia intensiva (UTI), com ventilação invasiva e não invasiva. Essas recomendações devem ser aplicadas a todos os profissionais diretamente envolvidos no procedimento (médicos, técnicos de enfermagem, tecnólogos etc.). Eco: ecocardiograma; ETE: ecocardiografia transesofágica; ETT: ecocardiografia transtorácica; RMC: ressonância magnética cardíaca; SPECT: tomografia computadorizada por emissão de fóton único; TCC: tomografia computadorizada cardíaca*.

**Figura 2:**
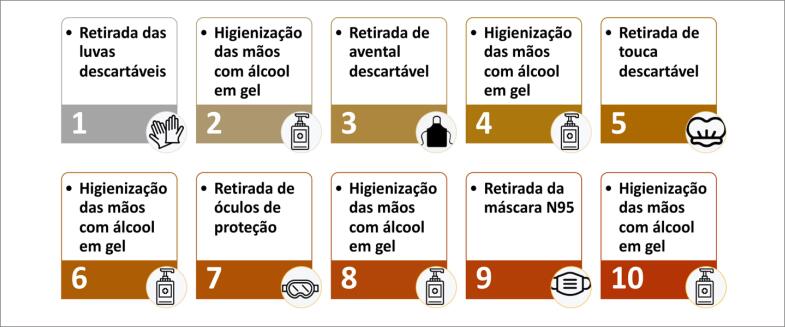
Fluxo de desparamentação com equipamentos de proteção individual.

### 2.4. Cuidados com Equipamentos

- Restringir o número de acessórios aos minimamente necessários à execução do exame para reduzir a necessidade de limpeza e desinfecção após o procedimento, o risco de contaminação e a transmissão cruzada.
- Considerar o uso de capa protetora envolvendo equipamento e transdutor (no caso de aparelhos de ultrassom), contanto que não dificulte o manuseio do aparelho e aumente o tempo de exame.
- Todos os equipamentos e acessórios devem ser limpos e desinfectados após cada uso de acordo com as diretrizes de desinfecção de equipamentos. Exames sob estresse e ETE ou aqueles feitos em pacientes com emissão de aerossóis (em unidade de terapia intensiva [UTI] ou sob ventilação invasiva ou não invasiva) necessitam de um intervalo de agendamento maior, porque a desinfecção será mais demorada. Protocolos de limpeza e desinfecção estão detalhados no Material Suplementar.

### 2.5. Cuidados Especiais para Exames sob Estresse

O exame sob estresse é essencial na avaliação de pacientes com doença coronariana suspeita ou confirmada. Isso inclui estresse com exercício ou estresse farmacológico com qualquer uma das imagens de modalidades de cardiologia nuclear [Tomografia computadorizada por emissão de fóton único (SPECT)/tomografia por emissão de pósitron (PET)], ecocardiografia ou ressonância magnética cardíaca (RMC). No entanto, o exame sob estresse físico pode aumentar o risco de contaminação por gotículas e deve ser adiado (em pacientes com baixo risco de COVID-19) ou não realizado (em pacientes com COVID-19 suspeita ou confirmada). Deve ser dada preferência ao estresse farmacológico. Na fase de recrudescimento da pandemia, quando clinicamente apropriado, o estresse sob exercício deve incluir precauções dicionais, tais como:^11^

- Conheça os padrões de circulação de ar do laboratório – comsulte a engenharia sobre equipamento/equipe otimizados. Dada a incerteza em relação à capacidade de gerar aerossóis durante o teste de esforço, é recomendável utilizar uma sala dedicada para teste de exercício, com pressão negativa, se possível.
- Evite a medição manual da pressão arterial, se possível. A medição de pressão arterial automatizada é comumente usada e razoavelmente precisa tanto em pacientes estacionários quanto em pacientes submetidos a estresse farmacológico.
- A equipe que supervisiona o teste deve manter distância (2 metros) do paciente sempre que possível.
- O pessoal envolvido deve incluir o uso de máscara facial (*face shield*) (particularmente durante ecocardiografia sob estresse com exercício) e luvas, além dos EPI comuns a todos os métodos.
- Quando possível, o paciente deve ser encorajado a usar uma máscara enquanto se exercita.
- Se o exercício for considerado necessário, considere rastrear a COVID-19 antes do teste de esforço.
- Deve ser feita uma escolha criteriosa do protocolo de exercício, pois os mais lentos aumentam o tempo de interação com o paciente. O protocolo de bicicleta está associado a ventilações de pico mais baixas por minuto.

### 2.6. Cuidados Especiais para Ecocardiografia Transesofágica

Dentre todas as modalidades ecocardiográficas, o estudo transesofágico (ETE) provavelmente é o que implica maior risco de contaminação da equipe, em função do manuseio das vias aéreas, do contato com secreções, da proximidade do examinador da boca do paciente e do estímulo da tosse que pode acontecer durante a passagem da sonda para o esôfago. Dessa forma, sua indicação deve ser avaliada criteriosamente, e recomenda-se o nível máximo de precaução para todos os exames, mesmo naqueles realizados no centro cirúrgico ou em pacientes que não são positivos ou suspeitos de COVID-19^2^^,^^3^^,^^8^^,^^12^^,^^13^ (ver Tabela 2). Idealmente, deve haver uma sala específica para ETE que precisará ter, além de proteção para o equipamento de ecocardiografia (isolamento ou cobertura com material impermeável) e para todo o material necessário para o procedimento, um protocolo estrito e demorado de desinfecção do ambiente entre os exames (cerca de 1 hora).

### 2.7. Cuidados Especiais para Ecocardiografia Pediátrica

Considerando a possibilidade aumentada de crianças assintomáticas ou infectadas com sintomas mínimos, as medidas de triagem aplicadas ao paciente adulto podem ser insuficientes, o que implica ajustes na realização da ecocardiografia ambulatorial ou hospitalar. O médico ecocardiografista deve ser preferencialmente o único em contato com a criança, no caso de uma criança ativa e cooperativa. Crianças menores de 2 anos de idade têm dificuldade para usar a máscara, o que leva a maior risco de exposição ao vírus. Além da principal forma de transmissão de SARS-CoV-2 por meio de gotículas respiratórias, a transmissão através de fômites é assumida como plausível.^14^ Portanto, troca de fraldas deve ser evitada, se possível, durante o exame, e, caso necessário, realizado com higiene adequada. Devido ao maior risco da forma assintomática da COVID-19 em crianças, alguns centros localizados em regiões endêmicas optam por testar as novas admissões pediátricas hospitalares para SARS-CoV-2.

### 2.8. Cuidados Especiais para Ecocardiografia Fetal

Nas instituições na qual a ecocardiografia fetal (EF) é realizada no setor de cardiologia, sugere-se que a gestante se mantenha em área separada do paciente pediátrico, tanto na sala de espera quanto na sala do procedimento. Diferentemente dos surtos virais anteriores (H1N1, SARS-CoV, MERS-CoV), que foram associados a complicações graves em gestantes, atualmente, as informações, embora limitadas, sugerem que as mesmas não são mais suscetíveis à infecção por SARS-CoV-2 ou, se infectadas, mais propensas a desenvolver complicações graves.^15^ No entanto, dada a incerteza e a possibilidade de aumento do risco à medida que mais dados se tornam disponíveis, os CDC alertam que é sempre importante que as mulheres grávidas se protejam da doença. Recomenda-se no máximo um acompanhante, que deve passar pelo mesmo processo de triagem da gestante; entretanto, o ideal é que a sala do ecocardiograma seja limitada a gestante e ao médico executor para minimizar a exposição.

## 3. Utilização da Ecocardiografia em Adultos na Era COVID-19

A ecocardiografia é o método de primeira linha no diagnóstico, avaliação prognóstica e orientação terapêutica em diversas doenças cardiovasculares. Durante o surto do novo coronavírus, permanece como método crucial de imagem, principalmente pela sua portabilidade em relação aos outros métodos de imagem, o que permite a realização à beira do leito em pacientes isolados e/ou críticos.^13^ Entretanto, como implica contato próximo entre o examinador e o paciente, traz alto risco de infecção por COVID-19.^10^

### 3.1. Priorização e Indicações de Ecocardiografia Transtorácica em Pacientes Adultos de Baixo Risco para COVID-19

Mesmo em pacientes com baixo risco de COVID-19, a reintrodução dos exames ambulatoriais deve ser progressiva, levando em consideração os critérios de prioridade e a fase da pandemia (ver Tabela 1).^10^^,^^11^^,^^13^ A seguir, estão descritas indicações de cada nível de prioridade.

#### 3.1.1. Exames de Alta Prioridade (Essenciais)

- Indivíduos com sintomas cardiovasculares agudos ou com piora recente. Exemplo: insuficiência cardíaca classe funcional (CF) III ou IV, síncope de provável origem cardíaca, dor torácica, arritmias, acidente vascular cerebral (AVC); suspeita de doença valvar aguda (regurgitação mitral ou aórtica); sintomas agudos em portador de prótese valvar; suspeita de estenose aórtica grave sintomática sem diagnóstico prévio.^7^
- Avaliação antes de terapia clínica urgente, mesmo em paciente assintomático. Exemplo: ecocardiograma basal antes do início de quimioterapia; avaliação da fração de ejeção do ventrículo esquerdo (FEVE) antes de implante de cardiodesfibrilador para prevenção primária.
- Planejamento de intervenção cardiovascular urgente: plastia de valva mitral, implante da valva aórtica transcateter (TAVI), oclusão de apêndice atrial esquerdo.
- Monitoramento da segurança de terapia clínica. Exemplo: seguimento da quimioterapia em paciente com alto risco de cardiotoxicidade, mesmo que assintomático.
- Controle após terapia cirúrgica ou invasiva recente. Exemplo: suspeita de derrame pericárdico após implante de dispositivo ou cirurgia cardíaca, mesmo que assintomático.
- Suspeita de endocardite infecciosa com alta probabilidade pré-teste.
- Suspeita de doença pericárdica ou de progressão de derrame pericárdico.
  Em pacientes internados, as indicações de ecocardiograma de urgência (ou alta prioridade) geralmente são as mesmas que antes da pandemia (tais como complicações mecânicas após infarto agudo do miocárdio, tamponamento, dissecção de aorta entre outras).

#### 3.1.2. Exames de Média Prioridade

- Monitoramento da progressão de doença crônica miocárdica ou valvar grave assintomática. Exemplo: cardiomiopatia, estenose aórtica, insuficiência mitral primária, prótese valvar disfuncionante.
- Surgimento de sintomas em pacientes com doença cardíaca ou pulmonar conhecida.
- Insuficiência cardíaca com FEVE reduzida (ICFEr), quando a FEVE determina terapia médica ou implante de dispositivo.
- Avaliação antes de procedimento ou terapia de rotina. Exemplo: cirurgia não urgente.
- Monitoramento do resultado de terapia. Exemplo: tratamento de cardiomiopatia dilatada em regressão, tratamento de rejeição após transplante cardíaco, Takotsubo (cardiomiopatia de estresse), doença de Kawasaki, disfunção de ventrículo direito (VD) após embolia pulmonar, pericardiocentese, avaliação de dispositivo de assistência ventricular.
- Avaliação inicial de novo sopro inexplicado.
  Esses exames podem ser reintroduzidos em áreas em que se observa um recrudescimento da pandemia.

#### 3.1.3. Exames de Baixa Prioridade

São exames eletivos, geralmente solicitados anualmente ou bianualmente, para seguimento de doenças crônicas assintomáticas ou sem mudança do estado prévio, em que o resultado do exame não mudará o tratamento e/ou o desfecho a curto prazo. Esses exames podem ser adiados para período de menor transmissibilidade ou quando as restrições tiverem sido suspensas ou flexibilizadas, especialmente se já há ecocardiograma prévio dos últimos 12 meses.^16^

### 3.2. Priorização e Indicações de Ecocardiografia Transtorácica em Pacientes Adultos com COVID-19 Suspeita ou Confirmada

Do ponto de vista cardiovascular, os pacientes acometidos pelo novo coronavírus (SARS-CoV-2) podem cursar com evidência de disfunção miocárdica (tanto esquerda quanto direita), alterações vasculares, arritmias, fenômenos tromboembólicos e derrame pericárdico.^4^ Dessa forma, a ecocardiografia também pode auxiliar no julgamento clínico de pacientes com COVID-19, em situações como:^2^^,^^3^^,^^17^^–^^20^

- Dispneia incapacitante. A dispneia é muito comum em pacientes com pneumonia secundária à COVID-19 (situação em que a troponina também eleva, podendo levar à falsa hipótese de miocardite). Nesse caso, um BNP normal (mesmo com troponina elevada) pode excluir a necessidade de ecocardiografia. Ultrassonografia de pulmão, em mãos experientes, pode auxiliar no diagnóstico diferencial entre insuficiência cardíaca (IC) e pneumonia.
- Pacientes com prévia doença cardíaca com mudança no estado hemodinâmico ou sinais e sintomas de envolvimento desproporcional do pulmão.
- Cardiomegalia na radiografia de tórax.
- Arritmias clinicamente significativas ou de início agudo.
- Dor torácica com alterações eletrocardiográficas e/ou elevação de troponinas. Se houver forte suspeita de miocardite e a ressonância magnética for indicada por ser considerada crucial para o tratamento em determinado caso, pode-se inicialmente prescindir da ecocardiografia.
- Instabilidade hemodinâmica, falência respiratória e/ou choque de etiologia incerta.
- Suspeita de hipertensão pulmonar e/ou disfunção ventricular direita.

Em janelas acústicas difíceis, o uso de contraste ecocardiográfico pode ser empregado para possibilitar a realização do exame, reduzir o tempo de realização, e evitar diagnóstico inadequado ou outros exames desnecessários.^21^^,^^22^

Nesses pacientes com COVID-19, o exame deve ser direcionado para a questão clínica. Ecocardiografia seriada deve ser evitada, a não ser que haja uma clara mudança no estado clínico (instabilidade hemodinâmica). Entretanto, na UTI, muitas vezes, o ecocardiograma é usado para monitorar a evolução de pacientes críticos, especialmente quanto ao manejo de fluidos. Nesse caso, um ultrassom focado ou a ecocardiografia direcionada podem ser utilizados. Tais protocolos são descritos a seguir.

### 3.3. Protocolos de Ecocardiografia Transtorácica em Pacientes Adultos na era COVID-19

#### 3.3.1. Ecocardiografia Direcionada

É aquele exame direcionado à questão clínica, porém completo o suficiente para abranger todas as hipóteses clínicas. Esse protocolo deve ser feito pelo ecocardiografista em indivíduos com COVID-19, sem monitoramento com eletrocardiograma . Deve incluir a avaliação dos seguintes parâmetros:^2^

- Ventrículo esquerdo: avaliação quantitativa da função sistólica global (fração de ejeção), sinais de disfunção regional, dimensões da cavidade.
- Ventrículo direito: função global, variação fracional de área (FAC) ou excursão sistólica do plano tricúspide (TAPSE), dimensão da cavidade, velocidade e gradiente da regurgitação tricúspide, se possível.
- Valvas: avaliação grosseira da anatomia e Doppler colorido, porém, se houver sinais de disfunção, aprofundar a avaliação.
- Pericárdio: presença de espessamento e/ou derrame.

#### 3.3.2. Ultrassom Focado – Indicações, Protocolos e Principais Achados

O ultrassom focado, também conhecido como *point of care* está sendo largamente utilizado como forma de suporte ao diagnóstico, manejo e acompanhamento seriado dos pacientes infectados por COVID-19.^23^ As grandes vantagens do ultrassom focado na COVID-19 incluem a sua ampla disponibilidade nos ambientes de urgência/emergências e terapia intensiva, elevada acurácia diagnóstica, vasta quantidade de informações clínicas que fornece e facilidade de utilização e realização à beira do leito – evitando o transporte de pacientes para a radiologia e a disseminação do vírus dentro do hospital. O exame pode ser realizado tanto com equipamentos de ultrassom convencionais, como equipamentos portáteis ou ultraportáteis (de bolso), sendo esses últimos preferíveis pela facilidade de desinfecção e uso à beira do leito. Todos os cuidados de proteção pessoal e para o equipamento descritos anteriormente devem ser adotados. Dentre as aplicações do ultrassom focado nos pacientes com COVID-19, destacamos a ultrassonografia pulmonar e o ultrassom cardíaco.

#### 3.3.3. Ultrassonografia Pulmonar e Pleural Focada

O ultrassom de pulmão consiste em uma alternativa ágil para avaliar o grau de envolvimento pulmonar e acompanhar o resultado de intervenções terapêuticas à beira do leito. A acurácia diagnóstica do ultrassom pulmonar (LUS) tem se mostrado similar à da tomografia computadorizada (TC) de tórax em pacientes com queixas respiratórias, tais como dispneia e hipoxemia (sensibilidade 85%; especificidade 93% – para pneumonia não COVID-19).^24^ Achados normais ao LUS também apresentam uma excelente correlação com TC de tórax livre de alterações parenquimatosas, tais como opacificações em vidro fosco. Dessa forma, o LUS apresenta um elevado valor preditor negativo, possibilitando seu uso para a estratificação de risco dos pacientes.^25^ Assim, além de atuar no diagnóstico e estratificação de risco inicial, o LUS está sendo amplamente utilizado no monitoramento dos pacientes críticos com quadro de síndrome respiratória aguda grave pelo SARS-CoV-2.^26^ O LUS ajuda na tomada de decisões sobre necessidade de ventilação pronada, oxigenação por membrana extracorpórea (ECMO) e desmame da ventilação mecânica na insuficiência respiratória aguda.^27^ É útil também para excluir outras patologias, como pneumotórax, que pode ocorrer em indivíduos com ventilação e pressão positiva.

**Figura 3:**
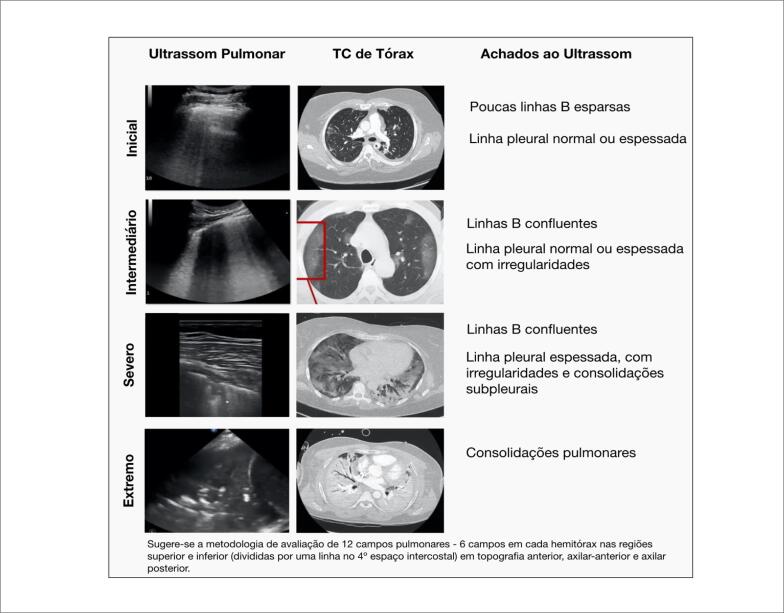
Achados do ultrassom pulmonar na COVID-19 e correlação com gravidade e achados tomográficos

Diversos protocolos de exame de LUS são descritos na literatura. No caso dos pacientes com COVID-19, recomendamos a avaliação de pelo menos seis campos pulmonares em cada hemitórax, nas regiões anterior, axilar anterior e axilar posterior, acima e abaixo da linha do 4° espaço intercostal. Como o acometimento pulmonar na COVID-19 é bilateral e multifocal, podendo não ser uniforme, ressaltamos a importância de não restringir a avaliação apenas em alguns pontos da parede torácica e recomendamos explorar todo o tórax.^28^^,^^29^ Os achados da LUS na COVID-19 acompanham a evolução e a extensão do acometimento pulmonar identificado pela TC de tórax e estão descritos na Figura 3. É importante ressaltar que derrame pleural não é um achado frequente da SARS-CoV-2, fato que ajuda a tornar pouco provável a infecção por COVID-19. Ao longo do processo de recuperação, a normalização do pulmão é gradual, sendo possível observar o desaparecimento das consolidações subpleurais, normalização da linha pleural e reaparecimento das linhas A.^30^

#### 3.3.4. Ultrassom Cardiovascular Focado

**Figura 4:**
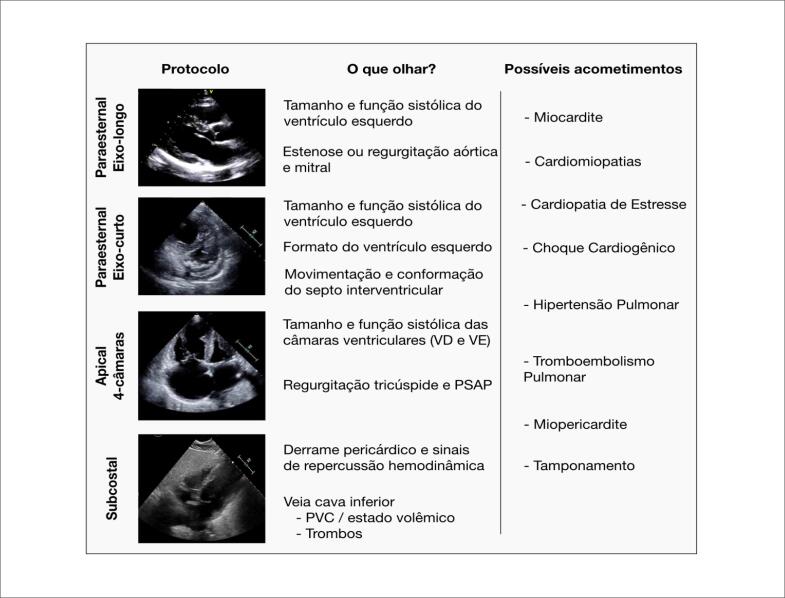
Protocolo do ultrassom cardiovascular focado. Janelas, principais achados e possíveis doenças. VD: ventrículo direito; VE: ventrículo esquerdo; PVC: pressão venosa central.

O ultrassom cardiovascular focado está sendo utilizado por médicos emergencistas ou intensivistas na linha de frente, para avaliação rápida e rastreamento de doenças cardíacas e vaculares preexistentes, bem como para identificação precoce de alterações miocárdicas relacionadas à COVID-19.^23^ O objetivo do ultrassom cardíaco é avaliar qualitativamente função sistólica do ventrículo esquerdo, tamanho e contratilidade do ventrículo direito, tamanho e colapsibilidade da veia cava inferior, anormalidades grosseiras valvares e derrame pericárdico^29^ (Figura 4). Em pacientes com COVID-19, o ultrassom está indicado na presença de troponina e ou peptídio natriurético tipo B (BNP) elevados, concomitante às alterações eletrocardiográficas ou hemodinâmicas, ou diante da suspeita de embolia pulmonar.^31^ Nesse caso, uma das grandes vantagens do ultrassom focado é poder reduzir a necessidade de um exame ecocardiográfico convencional, o que reduz a exposição da equipe médica e diminui a necessidade de descontaminação do equipamento ecocardiográfico, além de economizar EPI. Não equivale a um ecocardiograma, porém é capaz de confirmar ou excluir um diagnóstico específico, em uma rápida avaliação à beira do leito, facilitando decisões terapêuticas. Também pode triar pacientes que necessitem de exame ecocardiográfico. Por outro lado, se não houver treinamento adequado, há o risco de aquisição de imagens inadequadas que levem a diagnósticos falso-positivos ou falso-negativos. Com isso, podem ocorrer tratamentos desnecessários e, por vezes, prejudiciais, indicação de ecocardiogramas inapropriados ou retardo no tratamento. Dessa forma, o uso do ultrassom cardíaco focado deve ser baseado em protocolos institucionais, e é recomendável certificação interna de competência e análise constante de qualidade, tendo em vista que a metodologia para ensino e treinamento desse método é altamente variável.

O ultrassom focado integrado (cardíaco e pulmonar) é ideal para a caracterização adequada do estado volêmico, função subjacente dos ventrículos, monitoramento da resposta a fluidos e titulação do vasopressor no caso de suporte inotrópico. Na disfunção do VD, o ultrassom vascular focado para investigação de trombose venosa profunda (TVP), pode complementar a avaliação.

### 3.4. Priorização e Indicações para Ecocardiografia sob Estresse na Era COVID-19

Em pacientes de baixo risco para COVID-19 que enfrentam situações em que a indicação é apropriada e o adiamento não é possível ou não recomendável (p. ex., pré-operatório de cirurgia em paciente com câncer e probabilidade pré-teste alta de doença arterial coronariana obstrutiva), o ecocardiograma sob estresse farmacológico deve ser preferido, por não ser considerado gerador de aerossóis. Outra alternativa para investigação de casos selecionados de doença arterial coronariana crônica durante a pandemia é priorizar a realização de angiotomografia coronariana.^10^ Quando for alcançada uma prevalência muito baixa de COVID-19 na comunidade, a ecocardiografia sob exercício pode novamente se tornar a primeira escolha, mas requer cuidados adicionais de segurança, conforme previamente descrito.

### 3.5. Priorização e Indicações para Ecocardiografia Transesofágica em Pacientes Adultos na Era COVID-19

Preocupação especial existe com ETE, pois o risco de contaminação do equipamento e dos profissionais de saúde por gotículas e aerossóis é muito alto. Assim, o valor incremental de ETE sobre a ecocardiografia transtorácica (ETT) deve ser cuidadosamente analisado, caso a caso, em conjunto com o médico assistente, e, se possível, enquanto perdurar a pandemia, deve-se optar por métodos alternativos, especialmente em pacientes com COVID-19.^2^^,^^12^ Por exemplo, uma das principais indicações de ETE – a pesquisa de trombos intracavitários – pode, durante a vigência da pandemia, ser substituída pela angiotomografia, exame que tem menor potencial de contaminação da equipe. Obviamente, isso só será viável nos casos com possibilidade de transporte e com função renal preservada. Sempre que possível, o diagnóstico completo por meio de ETT deve ser tentado. Deve-se reservar ETE a situações de crítica importância em que possa haver mudança de conduta, especialmente em UTI ou centro cirúrgico, onde não é possível utilizar outro método, e deve-se focar em responder à questão clínica. Em locais em que está havendo o recrudescimento da epidemia, a reintrodução dos serviços de ETE para pacientes ambulatoriais deve seguir critérios estritos, e cada caso precisará de uma avaliação individual. Recomenda-se que a reintrodução das agendas ambulatoriais seja realizada baseando-se na prioridade de execução que deve considerar a posição prévia na fila de espera, indicação clínica, condição clínica (sintomático ou não) e o potencial impacto do exame na história clínica (p. ex., exames necessários para um agendamento de procedimento posterior).^2^^,^^12^^,^^13^ Cuidados adicionais de agendamento e segurança específicos para ETE são descritos na Tabela 2. Dentre as várias indicações de ETE, destacam-se, como alta prioridade:^32^

- Endocardite infecciosa com envolvimento valvar ou paravalvar.
- Dissecção de aorta tipo A (Stanford) em paciente instável (se paciente estável, preferir tomografia; se suspeita de insuficiência aórtica associada, avaliar em conjunto com ETT).
- Início de suporte circulatório mecânico.
- Infarto agudo do miocárdio com suspeita de complicações mecânicas não detectadas por ETT (defeito do septo interventricular, ruptura da parede livre do ventrículo esquerdo ou de músculo papilar).
- Disfunção de prótese mal definida à ETT ou tomografia.
- Monitoramento de instalação de ECMO venovenosa para tratamento de pneumonia por COVID.
- Avaliação intraoperatória de resultado de plastia mitral, miectomia septal ou para diagnóstico e manejo de complicações.
- Instabilidade hemodinâmica devido a choque indiferenciado em indivíduo com janela acústica inadequada à ETT (p. ex., período perioperatório de cirúrgica cardíaca; paciente sob ventilação pronada).
- Pesquisa de trombo em apêndice atrial esquerdo pré-cardioversão elétrica para restauração de ritmo sinusal, em paciente instável ou com tomografia computadorizada cardíaca (TCC) indisponível (TCC com baixa infusão de contraste é a primeira opção em pacientes com COVID-19).
  Não está recomendado a realização de ETE para:
- Pesquisa de endocardite infecciosa (EI) em paciente com febre transitória sem bacteremia ou novo sopro.
- Bacteremia transitória com identificação de patógeno não tipicamente associado a EI ou com fonte de infecção não endovascular documentada.
- Reavaliação de prévia imagem ecocardiográfica de vegetação em indivíduo estável quando nenhuma mudança de terapia é prevista.

## 4. Utilização da Ecocardiografia Pediátrica, Congênita e Fetal na Era COVID-19

O exame ecocardiográfico em crianças pode criar um risco aumentado de exposição para a equipe e a comunidade, tendo em vista que, apesar da menor ocorrência de doença grave, paradoxalmente, um grande número de crianças infectadas pode ser assintomático ou minimamente sintomático, além da necessidade da presença de acompanhante adulto, implicando ajustes para o atendimento especializado. Considerando que a população pediátrica e de portador de cardiopatia congênita é diferente da adulta, no que diz respeito ao risco de transmissão e indicações para ecocardiografia, o objetivo deste tópico é atualizar as indicações e protocolos para realização de ETT e de EF para essa população.

### 4.1. Priorização e Indicações de Ecocardiografia Transtorácica em Pacientes Pediátricos ou Congênitos

Na faixa etária pediátrica e nos portadores de cardiopatia congênita, as indicações absolutas incluem suspeita de cardiopatia congênita, seguimento pré-operatório e pós-operatório de cardiopatia congênita, crianças portadoras de cardiopatia adquirida, transplante cardíaco, crianças com risco aumentado de comprometimento da função cardíaca (tratamento com quimioterapia) e complicações cardíacas por infecção respiratória.^33^^,^^34^ Essas indicações deverão ser categorizadas como alta, média ou baixa prioridade, conforme demonstrado na Tabela 2, para a reintrodução dos exames em nível ambulatorial, de acordo com a repercussão hemodinâmica e o julgamento clínico.

### 4.2. Otimização do Protocolo de Ecocardiografia Transtorácica em Pacientes Pediátricos ou Congênitos

Recomenda-se que a ETT seja realizada da forma tradicional devido à grande variabilidade anatômica e aos desafios, no que diz respeito à avaliação da função sistólica e diastólica, reservando o estudo focado para utilização na unidade de emergência e terapia intensiva pediátrica, assim como nos pacientes com suspeita ou confirmação de COVID-19. No caso de exame potencialmente complicado, recomenda-se direcioná-lo ao ecocardiografista mais experiente, aumentando a probabilidade de realizar um exame adequadamente detalhado, preciso e rápido, sem necessidade de suporte prático adicional.

Recentemente, foi emitido um alerta acerca da síndrome inflamatória multissistêmica em crianças e adolescentes, descrita como uma apresentação clínica na maioria das vezes mais tardia e associada à COVID-19, caracterizada pela presença de manifestações clínicas similares às observadas na síndrome de Kawasaki típica, síndrome de Kawasaki incompleta e/ou síndrome do choque séptico.^35^ A faixa etária mais acometida é a escolar (idade média de 9 anos), critério que a diferencia da síndrome de Kawasaki, sendo os sintomas gastrointestinais predominantes. Há acometimento cardíaco com comprometimento da FEVE, descrita por diversos grupos, em praticamente 100% dos casos, choque cardiogênico e envolvimento das artérias coronárias que podem apresentar grau variável de dilatação, e em alguns grupos são descritos aneurismas. Os sintomas respiratórios são leves e pode haver erupção cutânea e envolvimento das mucosas.^35^^,^^36^

Na suspeita clínica da síndrome inflamatória multissistêmica em crianças e adolescentes, os principais objetivos da avaliação ecocardiográfica consistem em identificar possível dilatação do ventrículo esquerdo, medir a função sistólica por meio da FEVE, quantificar o grau de regurgitação valvar, avaliar o aspecto morfológico das artérias coronárias (dilatação e/ou aneurisma) e pericárdio.

### 4.3. Priorização e Indicações de Ecocardiografia Fetal

A avaliação por EF apresenta desafios, considerando-se que existe um período finito da gestação na qual é necessário o planejamento e a tomada de decisão perinatal e neonatal. A princípio, dependendo do grau de estratificação de risco, considerou-se nos casos de gestantes de baixo risco a não realização de EF; no grupo de risco moderado, adiar a EF para uma data posterior quando o risco de SARS-CoV-2 estiver diminuído ou após 28 semanas de gestação; e no grupo de alto risco, agendar e realizar o exame rapidamente.^15^ Entretanto, com o prolongamento da pandemia, considerando que a gravidez continuará e que as doenças cardíacas fetais podem ser críticas, recomenda-se que a EF seja realizada seguindo as recomendações das diretrizes previamente publicadas.^37^^,^^38^ A execução deve ser realizada conforme os protocolos de EPI à gestante e ao profissional de saúde. A avaliação ecocardiográfica deve ser a mais completa possível, evitando-se reavaliações.

A possibilidade de infecção pré-natal ou perinatal deve ser considerada quando os neonatos são transferidos para a unidade de terapia neonatal pediátrica ou cardíaca após o parto. Os dados sobre a transmissão vertical do SARS-CoV-2 ainda são escassos;^39^^,^^40^ no entanto, se a puérpera testar positiva para o vírus dentro de 14 dias após o parto, o recém-nascido deve ser também testado e tratado como positivo, até que um resultado negativo seja confirmado.

A prestação de serviços ecocardiográficos à população pediátrica e portadores de cardiopatia congênita permanece crucial durante a pandemia de SARS-CoV-2 e devemos continuar os cuidados a esse grupo de pacientes sempre minimizando os riscos para a equipe profissional de saúde, pacientes e o público.

### 4.4. Priorização e Indicações da Ecocardiografia Transesofágica em Pacientes Pediátricos

Em pacientes portadores de cardiopatia congênita, a ETE é considerada parte integrante do cuidado intraoperatório, assim como durante as intervenções hemodinâmicas. Nos casos ambulatoriais frente ao risco alto de exposição ao SARS-CoV-2, recomenda-se adiar ou substituí-lo por uma modalidade de imagem alternativa, como, por exemplo, a realização de ETT associada à injeção de contraste salino agitado, TCC e RMC contrastadas. Nas crianças, sempre devemos equilibrar os riscos e benefícios que podem ocorrer em procedimento de aerossol com o risco de transporte, necessidade de desinfecção da sala de TCC ou RMC, administração de contraste ou radiação na TCC, além do tempo mais prolongado para realização de RMC.

### 4.5. Protocolo para Realização da Ecocardiografia Transesofágica em Pacientes Pediátricos

Devido à falta de confiabilidade dos sintomas para prever o *status* da COVID-19 em crianças, foi proposta recomendação específica para realização de ETE em crianças:^15^

1. Todos os pacientes na faixa etária pediátrica devem ser considerados positivos para realização de ETE, a menos que tenham um teste COVID-19 negativo dentro de 48 a 72 horas. Se teste COVID-19 negativo, a ETE poderá ser realizada usando as precauções-padrão (luvas, máscara e proteção para os olhos).
2. Em pacientes pediátricos sem teste negativo para COVID-19 dentro 72 horas, intubados antes da chegada ao centro cirúrgico, o risco de aerossolização é considerado baixo. A introdução da sonda pode ser realizada pelo anestesista ou ecocardiografista, de acordo com os procedimentos e precauções-padrões da instituição.
3. Em pacientes assintomáticos sem teste negativo para COVID-19 dentro de 72 horas que requerem intubação no centro cirúrgico, recomenda-se que seja realizada pelo anestesista usando EPI apropriados e respiradores purificadores de ar. Esse processo deve ser seguido por um período de espera de aproximadamente 20 a 30 minutos, dependendo dos protocolos locais e dos fatores ambientais, permitindo a completa troca de ar na sala. Nesse período, não deve ser permitido o trânsito de pessoas na sala. A recomendação é que a sonda da ETE seja introduzida pelo anestesista imediatamente após a estabilização das vias aéreas para minimizar o risco de exposição de outras pessoas. Após o período de espera, a manipulação da sonda pode ser realizada pelo ecocardiografista, de acordo com os procedimento e precauções padronizadas.
4. Para crianças COVID-19 positivas ou sintomáticas, o isolamento estrito é mandatório. Deve-se considerar fortemente a introdução da sonda pelo anestesista para minimizar o risco de exposição de outros profissionais. A equipe de profissionais de saúde do centro cirúrgico, sala de recuperação ou sala de procedimentos deve usar equipamento de isolamento rigoroso durante todo o tempo e estar treinada para vestir e retirar EPI. Somente o pessoal essencial é permitido no centro cirúrgico para atenuar o risco de exposição (apenas uma pessoa de eco) e preservar EPI.

## 5. Utilização da Ultrassonografia Vascular na Era COVID-19

Uma das situações que requer maior cuidado nos pacientes infectados por SARS-CoV-2 é o desenvolvimento de coagulopatia, caracterizada, em grande parte, por tendência à trombose dos sistemas venoso e arterial, bem como microvasculatura. Klok et al.^31^ avaliaram a incidência de tromboembolismo venoso (TEV) e complicações trombóticas arteriais em 184 pacientes com COVID-19 internados em UTI. A despeito da profilaxia para TEV realizada em todos os pacientes, foi detectada 31% de incidência de complicações trombóticas.^31^ Por esse motivo, durante a pandemia, houve um aumento da demanda na ultrassonografia vascular (USV), em especial a USV venosa, nos pacientes com COVID-19, a maioria deles internados. Por esse motivo, discussão mais aprofundada será feita a seguir para esse exame.

### 5.1. Priorização e Indicações de Ultrassonografia Vascular em Pacientes de Baixo Risco para COVID-19

Em pacientes ambulatoriais sem COVID-19, é necessário subdividir os tipos de exames e categorizá-los de acordo com a prioridade da indicação (ver Tabela 1). São considerados exames de alta prioridade (essenciais), a USV venosa para pesquisa de TVP e a USV de carótidas e vertebrais em pacientes com suspeita de AVC. Na doença obstrutiva periférica, a prioridade da USV arterial dependerá da indicação do tratamento cirúrgico.

Demais exames devem ser categorizados como média ou baixa prioridade a critério do médico assistente, desde que não haja indicação de procedimento invasivo de urgência. Nesse contexto, pode-se incluir a USV de aorta e ramos, a USV para mapeamento venoso de varizes e a USV de carótidas para rastreamento de doença obstrutiva carotídea no pré-operatório de cirurgia cardíaca.

### 5.2. Priorização e Indicações da Ultrassonografia Vascular em Pacientes com COVID-19

- Suspeita de tromboembolismo pulmonar (TEP): a USV tem baixa acurácia no diagnóstico de TEP, porém poderá ser indicada quando houver alto risco de sangramento, quando o resultado mudar a conduta ou quando a suspeita de TEP for alta e não houver angiotomografia disponível.^41^
- Suspeita de TVP: nos indivíduos com alta suspeita clínica de TVP e com risco de sangramento elevado, a USV do sistema venoso de membros está indicada.^41^
- Suspeita de obstrução arterial aguda de membros superiores ou membros inferiores (USV arterial).
- AVC de causa a esclarecer (USV de carótidas e vertebrais).

### 5.3. Situações em que a Ultrassonografia Vascular Não Está Recomendada para Pacientes com COVID-19

A USV venosa não deve ser utilizada como marcador para alterar o planejamento da terapia com anticoagulantes proposta para o paciente.

Exames laboratoriais não são indicadores da necessidade de realização do exame; dessa forma, um valor alto de D-Dímero não justifica a realização da USV para investigação de TVP. Já em casos com D-Dímero negativo, não há necessidade da realização da USV para pesquisa de TVP.

A TVP nos membros superiores tem baixa morbidade em pacientes criticamente enfermos, portanto, não há recomendação de rotina para a realização da USV venosa dessa região.

Concluindo, a USV não está recomendada em quaisquer situações em que o resultado do exame não determine mudança de conduta ou não seja um pré-requisito para cirurgia de urgência.

### 5.4. Otimização dos Protocolos de Ultrassonografia Vascular

O protocolo de USV venoso completo é o mais recomendado para a investigação de TVP; no entanto, a compressão de três ou dois pontos (*point of care*) em pacientes internados criticamente enfermos com COVID-19 parece ser uma opção razoável, exceto se a queixa de dor do paciente for no segmento infrapatelar (nesse caso, deve-se fazer o protocolo completo). O protocolo de três pontos avalia a compressibilidade de todas as veias proximais do membro inferior investigado. O protocolo de dois pontos avalia a compressibilidade na veia femoral comum 1 a 2 cm acima e abaixo da junção safenofemoral (na prega inguinal) e na veia poplítea até onde há a confluência das veias da perna.^42^ A ausência total ou parcial da compressibilidade da veia acometida, bem como a dilzatação do vaso devido à presença do trombo intraluminal detectada pelo modo bidimensional, são sinais ultrassonográficos de TVP. A sensibilidade da compressão de três pontos é considerada maior que o protocolo de compressão de dois pontos (90,57% *vs*. 82,76%) com especificidade semelhante (98,52%).^43^

Além de demandar um tempo menor de exposição do examinador, o protocolo de USV *de point of care* pode ser realizado pelo médico emergencista desde que haja um treinamento prévio. Para a fase pós-pandemia, sempre que possível, deve-se dar preferência para realizar o protocolo completo.

Os demais exames de USV, tais como USV de carótidas e vertebrais, USV arterial de membros, quando se fizerem necessários, seguirão os protocolos de execução já estabelecidos.^44^ Todas as medidas de proteção e uso de EPI devem ser seguidas conforme previamente discutido.

## 6. Utilização da Ressonância Magnética Cardíaca na Era COVID-19

### 6.1. Priorização e Indicações

A pandemia da COVID-19 ocasionou uma importante redução no número de solicitações ambulatoriais de RMC, sendo realizados apenas aqueles exames de alta prioridade, tais como na suspeita clínica de miocardite e diagnóstico diferencial de massas cardíacas, bem como outras situações de exceção como avaliação de arritmias ventriculares complexas.

A RMC está bem-definida como um método de excelência na avaliação fidedigna da função ventricular e volumes cardíacos, bem como de isquemia, viabilidade miocárdica, detecção de áreas de fibrose miocárdica, estudo de doenças infiltrativas e de depósito, avaliação estrutural do paciente com arritmias cardíacas e em casos específicos, complementando a avaliação ecocardiográfica nas doenças valvares e nas cardiopatias congênitas. Em pacientes com baixo risco de COVID-19, a priorização para realização deve seguir o racional apresentado na Tabela 1. A sua realização tem a vantagem de em um único exame obter uma avaliação global do coração, oferecendo múltiplas informações funcionais e estruturais, evitando a ida do paciente ao hospital ou centro médico para realizar diversos exames, diminuindo a circulação do paciente e otimizando recursos.^45^Em pacientes com COVID-19, a RMC fornece informações acuradas no diagnóstico das miocardites, perimiocardites, infarto agudo do miocárdio sem doença coronariana obstrutiva (MINOCA), quadros de Takotsubo e na diferenciação de quadros isquêmicos de inflamatórios. Contudo, a sua realização na fase aguda deve ser criteriosamente avaliada quanto ao risco de transmissibilidade e grau de instabilidade do paciente, podendo ser postergada, sempre que possível, para quando forem atingidos os critérios de cura. Por outro lado, um estudo recente que avaliou lesão miocárdica em pacientes recuperados de COVID-19 evidenciou alta prevalência de padrão de realce tardio não isquêmico e função sistólica preservada, já sem evidência de edema, sugerindo dano miocárdico permanente.^46^ A RMC pode ser, portanto, uma ferramenta importante para uma melhor compreensão dos mecanismos da lesão miocárdica e para avaliação da extensão do dano miocárdico após a recuperação.

### 6.2. Otimização de Protocolos de Ressonância Magnética Cardíaca

Os protocolos de RMC devem ser reduzidos e focados (máximo de aproximadmente 30 minutos) em todas as indicações clínicas; o foco deve ser a avaliação da função miocárdica através das sequências de cine-RM; e a caracterização tecidual deve ser feita por meio do realce tardio miocárdico com gadolínio. A realização de uma sequência anatômica ponderada em T2 poderá ser realizada na tentativa de detectar edema miocárdico nos casos de suspeita de processo inflamatório agudo do miocárdio. Havendo disponibilidade, os mapas de T1 e T2 e/ou T2* associados às sequências de cine-RM e realce tardio constituem um protocolo otimizado e eficiente.^11^ Na avaliação de isquemia miocárdica, a aquisição de sequências de perfusão sob estresse farmacológico deve ser o foco, adicionando-se sequências de cine e realce tardio após o estresse. A RMC também pode ser empregada para o diagnóstico de trombos intracavitários, com um estudo rápido e direcionado com o uso do realce tardio com gadolínio, evitando a necessidade de ETE e reduzindo assim a exposição do ecocardiografista. A avaliação de massas cardíacas é realizada com alta sensibilidade pela RMC, podendo diferenciar características de benignidade ou malignidade e bastante limitada no diagnóstico de vegetações devido às suas pequenas dimensões associadas à característica mobilidade. A caracterização de massas deverá seguir o protocolo de rotina para este fim, utilizando-se as sequências de cine-RM, sequências anatômicas ponderadas em T1 com e sem saturação de gordura, T2, perfusão em repouso e realce tardio, sempre focadas na localização da massa. Doenças congênitas poderão ter sua avaliação otimizada com o uso de angioressonância 3D associadas a sequências de cine-RM, e, na eventual necessidade de avaliação valvar complementar ao ecocardiograma, é necessário priorizar a avaliação da função ventricular pela cine-RM e os protocolos direcionados para os aparelhos valvares com as sequências de mapeamento de fluxo.^47^

## 7. Utilização da Tomografia Computadorizada Cardíaca na Era COVID-19

### 7.1. Priorização e Indicações

A TCC pode ser utilizada na avaliação de múltiplas formas de doenças cardíacas em todas as fases da pandemia da COVID-19 de maneira rápida, eficiente e segura.^48^ Para tanto, a depender da fase da pandemia em que a região se encontre, é preciso balancear o risco da exposição ao vírus decorrente da realização do exame, com o benefício de seu resultado para a conduta e o tratamento.^11^ A Tabela 3 propõe uma priorização de indicações durante a pandemia pela COVID-19.

**Tabela 3:** Priorização das indicações da TCC durante a pandemia pela COVID-19

	Indicações de baixa prioridade	Indicações de média prioridade	Indicações de alta prioridade (considere realizar o exame nas próximas horas ou em até 2 a 4 semanas)
DAC	Escore de cálcio em assintomáticos		Dor torácica aguda com suspeita suficiente para DAC
	Dor torácica estável sem alta suspeita para DAC		Dor torácica estável com alto risco de eventos, ou quando se suspeita de anatomia de alto risco
Doença estrutural cardíaca	Pacientes com doença estrutural cardíaca estável (TAVI, TMVR, oclusão de AAE em conjunto com o Heart Team)	Pacientes necessitando de intervenção estrutural urgente (TAVI, TMVR, oclusão de AAE)
Fibrilação atrial	Avaliação de veias pulmonares para planejamento de ablação de fibrilação atrial	Avaliação do apêndice atrial esquerdo em arritmia atrial crônica antes de restauração do ritmo sinusal	Avaliação do apêndice atrial esquerdo em arritmia atrial aguda antes de restauração do ritmo sinusal
Insuficiência cardíaca	Investigação de DAC como causa de insuficiência cardíaca em pacientes com cardiomiopatia estável		Paciente internado com cardiomiopatia aguda e probabilidade baixa ou intermediária para DAC em que TCC pode mudar a conduta
			Avaliação de disfunção de dispositivo de assistência ventricular esquerda
Valvar	Avaliação da importância de estenose aórtica	Disfunção subaguda ou crônica de prótese valvar	Disfunção aguda e sintomática de prótese valvar, endocardite, extensão perivalvar de endocardite ou possível abscesso valvar
Massas/ congênito	Massas cardíacas suspeitas benignas ou improváveis de necessitar de biópsia ou planejamento cirúrgico		Novas massas cardíacas suspeitas de ser malignas ou com necessidade de biópsia ou planejamento cirúrgico
	Avaliação eletiva de anatomia congênita		Afastar trombo no ventrículo esquerdo após ecocardiograma inconclusivo, quando métodos alternativos (ressonância) não estão disponíveis

*DAC: doença arterial coronariana; TAVI: implante da valva aórtica transcateter; TMVR: implante da valva mitral transcateter; AAE: apêndice atrial esquerdo; TCC: tomografia computadorizada cardíaca*.

Quanto às indicações, as principais características diagnósticas e prognósticas da TCC devem ser levadas em consideração no momento da decisão:^49^

- A habilidade da TCC em excluir com precisão doença arterial coronariana (DAC) de alto risco pode prevenir admissões e utilização de recursos, além de indicar a hospitalização naqueles nos quais DAC de alto risco for detectada, principalmente em pacientes relutantes à procura de atendimento de urgência.
- O papel fundamental da TCC na avaliação anatômica pré-procedimento em doenças estruturais cardíacas, reduzindo o risco de complicações agudas e crônicas associadas às intervenções.
- Pode-se preferir a TCC à ETE para descartar trombos no apêndice atrial esquerdo e intracavitários, antes de cardioversão, reduzindo o risco de tosse e aerolização relacionadas com ETE.
- Nos pacientes suspeitos de infecção pela COVID-19 e nos confirmados, o benefício da TCC na maioria dos cenários clínicos deve ser menor que o risco de exposição e infecção dos funcionários. É necessário avaliar caso a caso.

## 8. Utilização da Cardiologia Nuclear na Era COVID-19

A cardiologia nuclear tem uma sólida base de conhecimentos de experiência clínica, tanto quanto seu valor diagnóstico e prognóstico. Todos os procedimentos usados nessa área têm a vantagem de utilizar protocolos e máquinas amplamente automatizados, permitindo um tempo menor de contato dos profissionais com o paciente. Além disso, à exceção de cintilografia miocárdica de exercício e cintilografia de ventilação perfusão, todos os demais métodos de medicina nuclear são não aerossolizantes.^50^ Isso pode minimizar a exposição ao vírus e reduzir a propagação de infecção, bem como conservar recursos preciosos.^51^

### 8.1. Indicações e Priorizações

A cardiologia nuclear tem um papel vantajoso no meio peripandêmico em pacientes sem COVID-19. O racional para a priorização de realização deve ser o mesmo descrito previamente na Tabela 2. Dentre as indicações, destacam-se:^11^

- Avaliação de isquemia em pacientes com DAC conhecida.
- Avaliação de pacientes com síndromes de dor no peito. É particularmente útil em pacientes que não são bons candidatos para imagens anatômicas não invasivas (p. ex., pacientes com *stents*, calcificação coronária, alergia a contraste, risco de agravamento da função renal).
- Avaliação da viabilidade miocárdica.
- Triagem para amiloidose.
- Identificação dos estágios inflamatórios da sarcoidose.
- Identificação de infecções em dispositivos implantados.

Por outro lado, estudos de cardiologia nuclear geralmente não são necessários no tratamento de doenças cardíacas agudas nos pacientes positivos para COVID-19. Os exames de ventilação ou com estresse físico devem ser omitidos em comunidades que se encontram no pico da pandemia e/ou em qualquer paciente com infecção conhecida ou suspeita por COVID-19, pelo alto risco de emissão de aerossóis.^52^ A seguir, são descritas as considerações específicas a respeito do protocolo de cintilografia de perfusão-ventilação.

### 8.2. Cintilografia de Perfusão-ventilação

O atual padrão-ouro para descartar embolia pulmonar (EP) em pacientes com COVID-19 é uma angiotomografia pulmonar (angio-CTP). No entanto, em pacientes com contraindicações para meios de contraste iodados, a angio-CTP não pode ser usada para descartar EP. Uma alternativa potencial é a SPECT pulmonar de perfusão usando albumina macroagregada (MAA) marcada com ^99m^Tc. Devido ao alto risco de produção de aerossóis associado à cintilografia de ventilação (aerossóis marcados com ^99m^Tc), a Sociedade Norte-Americana de Medicina Nuclear desencorajou o uso da combinação clássica de imagens de perfusão-ventilação em pacientes com COVID.^53^ Os exames de ventilação devem ser omitidos em qualquer paciente com infecção conhecida ou suspeita por COVID-19; portanto, foi proposto o uso da cintilografia pulmonar de perfusão, associada à tomografia de tórax ou radiografia de tórax.^54^

Para otimização dos protocolos em época de pandemia, deve-se aderir as boas práticas de imagem que podem tornar o procedimento seguro e eficiente nesse período, como descrito previamente, em especial:

- Usar protocolos que minimizem o tempo de estudo sem afetar a precisão do teste; por exemplo, apenas imagens de estresse quando for indicado.
- Evitar protocolos que podem aerossolizar – dar preferência ao estresse farmacológico em vez de exercício quando for possível.

**Figura 5:**
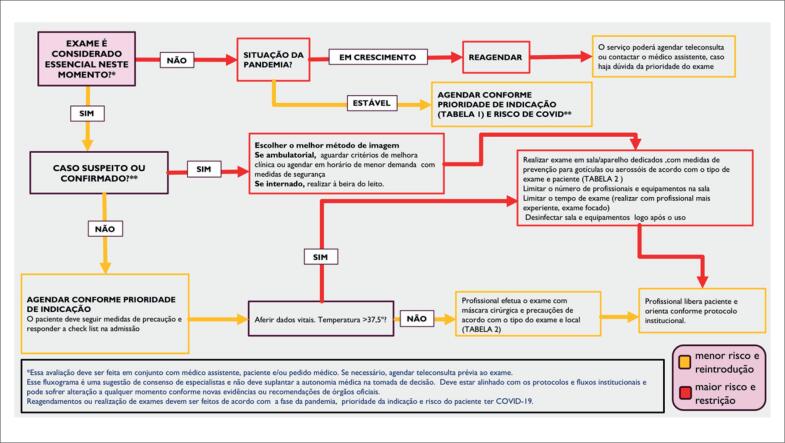
Fluxograma para reintrodução de exames de imagem cardiovascular na era da Covid-19

A Figura 5 é uma proposta que resume o protocolo geral para a reintrodução de exames de imagem cardiovascular. Em primeiro lugar, é necessário definir se o exame é essencial naquele momento. Se for essencial (urgente ou alta prioridade), definir se o paciente é ou não de alto risco para COVID e, em seguida, qual método implicará menor risco de exposição mesmo com o uso de EPI. Se o exame não for essencial/urgente, avaliar em que fase de pandemia a região se encontra. Se o local está no pico da pandemia, postergar os de menor prioridade. Se a região está em desaceleração da pandemia, reintroduzir o agendamento dos exames de acordo com o risco para COVID e prioridade da indicação, dentro de critérios de uso apropriado; por fim, quais EPI e fluxos devem ser utilizados de acordo com o risco de COVID e o exame escolhido. As Tabelas 1, 2 e 3 resumem a priorização das indicações, os protocolos de segurança e EPI que devem ser utilizados de acordo com o tipo de exame e o diagnóstico ou não de COVID. Dentre os exames de imagem cardiovascular, a ecocardiografia é o método de primeira linha. No entanto, tendo em mente a necessidade de minimizar a exposição do ecocardiografista ao novo coronavírus (SARS-COV-2) e usar racionalmente os recursos disponíveis, há situações em que métodos alternativos à ecocardiografia podem responder à questão clínica, especialmente em pacientes estáveis, com sintomas duvidosos ou encaminhados para realizar outros exames de imagem.^8^

## 9. Conclusão

A pandemia por COVID-19 nos forçou a reconsiderar melhor a realização de exames de imagem cardiovascular. Adaptações e mudanças foram necessárias devido ao impacto mundial desencadeado pela pandemia. O restabelecimento da “normalidade” dos serviços de imagem cardiovascular deverá ser progressivo e adaptado às diferenças regionais do país. Diante do impacto da doença cardiovascular na morbidade e mortalidade da população, não é possível negligenciar os sinais e sintomas cardiovasculares. Dessa maneira, mesmo em uma fase de pandemia, em que todas as atenções estão voltadas para o combate à COVID-19, pacientes e médicos devem ser encorajados a proceder à investigação cardiovascular, assegurando-os de que isso será realizado em ambiente seguro. Tal posicionamento reflete a opinião de especialistas baseada em diretrizes nacionais e internacionais e evidências científicas disponíveis até o momento, pois o conhecimento sobre a COVID-19 está em constante evolução. Nesse cenário, recomendações podem nos orientar a proteger pacientes e profissionais sem comprometer a assistência. Aliado a essas recomendações, o constante diálogo entre médicos imaginologistas, equipe clínica e pacientes constitui a melhor e mais eficiente conduta de enfrentamento da pandemia por COVID-19.
