# Supplementary material for: Posicionamento sobre Indicações e Reintrodução dos Métodos de Imagem Cardiovascular de Forma Segura no Cenário da COVID-19 – 2021
Source: Arq Bras Cardiol. 2021 Mar 3;116(3):659–78. [Article in Portuguese] doi: 10.36660/abc.20210133 (PMC8159553; doi:10.36660/abc.20210133)
Supplement: Translation

# Translation: Position Statement on Indications and the Safe Reintroduction of Cardiovascular Imaging Methods in the COVID-19 Scenario – 2021

**Authors:** Adenalva Lima de Souza Beck, Silvio Henrique Barberato, André Luiz Cerqueira de Almeida, Claudia R. Pinheiro de Castro Grau, Marly Maria Uellendahl Lopes, Ronaldo de Souza Leão Lima, Rodrigo Júlio Cerci, Ana Cristina Lopes Albricker, Fanilda Souto Barros, Alessandra Joslin Oliveira, Edgar Bezerra de Lira, Marcelo Haertel Miglioranza, Marcelo Luiz Campos Vieira, José Luiz Barros Pena, Tânia Mara Varejão Strabelli, David Costa de Souza Le Bihan, Jeane Mike Tsutsui, Carlos Eduardo Rochitte

**Affiliations:** 1 BrasíliaDF Brazil Instituto de Cardiologia do Distrito Federal – Fundação Universitária de Cardiologia, Brasília, DF – Brazil; 2 BrasíliaDF Brazil Hospital Sírio-Libanês, Brasília, DF – Brazil; 3 CuritibaPR Brazil CardioEco – Centro de Diagnóstico Cardiovascular, Curitiba, PR – Brazil; 4 CuritibaPR Brazil Quanta Diagnóstico e Terapia, Curitiba, PR – Brazil; 5 Feira de SantanaBA Brazil Santa Casa de Misericórdia de Feira de Santana, Feira de Santana, BA – Brazil; 6 São PauloSP Brazil Instituto do Coração (InCor) do Hospital das Clínicas da Faculdade de Medicina da Universidade de São Paulo (HCFMUSP), São Paulo, SP – Brazil; 7 São PauloSP Brazil Grupo Fleury, São Paulo, SP – Brazil; 8 São PauloSP Brazil Universidade Estadual Paulista (Unesp), São Paulo, SP – Brazil; 9 São PauloSP Brazil Diagnósticos da América SA (Dasa), São Paulo, SP – Brazil; 10 Rio de JaneiroRJ Brazil Universidade Federal do Rio de Janeiro (UFRJ), Rio de Janeiro, RJ – Brazil; 11 Belo HorizonteMG Brazil Instituto Mineiro de Ultrassonografia (IMEDE), Belo Horizonte, MG – Brazil; 12 VitóriaES Brazil Vascular Vitória S/C LTDA., Vitória, ES – Brazil; 13 São PauloSP Brazil Hospital Israelita Albert Einstein, São Paulo, SP – Brazil; 14 Porto AlegreRS Brazil Prevencor – Hospital Mãe de Deus, Porto Alegre, RS – Brazil; 15 Porto AlegreRS Brazil Instituto de Cardiologia do Rio Grande do Sul – Fundação Universitária de Cardiologia, Porto Alegre, RS – Brazil; 16 Belo HorizonteMG Brazil Faculdade Ciências Médicas de Minas Gerais, Belo Horizonte, MG – Brazil; 17 Belo HorizonteMG Brazil Hospital Felício Rocho, Belo Horizonte, MG – Brazil; 18 São PauloSP Brazil Instituto Dante Pazzanese de Cardiologia, São Paulo, SP – Brazil; 19 São PauloSP Brazil Hospital do Coração (HCor), São Paulo, SP – Brazil; 20 Rio de JaneiroRJ Brazil Hospital Pró-Cardíaco, Rio de Janeiro, RJ – Brazil

**Table:** Declaration of potential conflict of interests of authors/collaborators of the Position Statement on Indications and the Safe Reintroduction of Cardiovascular Imaging Methods in the COVID-19 Scenario – 2021

If, within the last 3 years, the author/collaborator of the statement:							
Names of statement collaborators	Participated in clinical and/or experimental studies sponsored by pharmaceutical or equipment companies related to this statement	Spoke at events or activities sponsored by industry related to this statement	Was (is) a member of a board of advisors or a board of directors of a pharmaceutical or equipment industry	Participated in normative committees of scientific research sponsored by industry	Received personal or institutional funding from industry	Wrote scientific papers in journals sponsored by industry	Owns stocks in industry
Adenalva Lima de Souza Beck	No	No	No	No	No	No	No
Alessandra Joslin Oliveira	No	No	No	No	No	No	No
Ana Cristina Lopes Albricker	No	No	No	No	No	No	No
André Luiz Cerqueira de Almeida	No	No	No	No	No	No	No
Carlos Eduardo Rochitte	No	No	No	No	No	No	No
Claudia R. Pinheiro de Castro Grau	No	No	No	No	No	No	No
David Costa de Souza Le Bihan	No	No	No	No	No	No	No
Edgar Bezerra de Lira Filho	No	No	No	No	No	No	No
Fanilda Souto Barros	No	No	No	No	No	No	No
Jeane Mike Tsutsui	No	No	No	No	No	No	No
José Luiz Barros Pena	No	No	No	No	No	No	No
Marcelo Haertel Miglioranza	No	No	No	No	No	No	No
Marcelo Luiz Campos Vieira	No	No	No	No	No	No	No
Marly Maria Uellendahl Lopes	No	No	No	No	No	No	No
Rodrigo Júlio Cerci	No	No	No	No	No	No	No
Ronaldo de Souza Leão Lima	No	No	No	No	No	No	No
Silvio Henrique Barberato	No	No	No	No	No	No	No
Tânia Mara Varejão Strabelli	No	No	No	No	No	No	No

## Cover Letter

The objective of this position statement is to inform clinicians, cardiologists, and medical imaging specialists on the recommended procedures, flows, and protocols for the duration of the SARS-CoV-2 pandemic aiming at a more efficient protection of health care professionals and patients. Recommendations are based on the best available scientific evidence at the moment and on the consensus among experienced specialists. Since the first case of the new coronavirus disease (COVID-19) in Brazil, many changes in recommendations and a large scientific debate regarding some of them have occurred within various sources of scientific knowledge. This is due to our currently incomplete knowledge on COVID-19, including its pathophysiological processes and aspects of SARS-CoV-2 transmission. Another reason for a variability in recommendations is related to the epidemiological phase of the pandemic in each region of the country. Notably, in a country with continental dimensions such as Brazil, the pandemic can reach different transmission phases in a specific moment, thus requiring particular measures for each region and phase of the pandemic.

In the beginning of the pandemic, a phase of great attention and lack of knowledge on what the future held, the Cardiovascular Imaging Department (DIC) of the Brazilian Society of Cardiology (SBC) published a brief and essential version of a position statement. The document provided rapid and practical fundamental guidance regarding safety procedures during non-invasive cardiovascular imaging procedures. This document was absolutely essential at that moment for providing the best possible protection for health care professionals. After the publication of a summary document in the initial stage of the pandemic, DIC-SBC and its specialists have deemed timely to update that position statement (now published in the form of an official scientific publication); this should include not only a much wider view of the procedures in light of the data acquired since then, but also a customized orientation for each epidemiological phase of the pandemic that could be useful for professionals working in non-invasive cardiovascular imaging for the next months or possibly years of coexisting with COVID-19.

## 1. Introduction

In view of the new coronavirus disease (COVID-19) pandemic and its high transmissibility, an urgent reorganization of cardiovascular imaging services was necessary for minimizing exposure to the severe acute respiratory syndrome coronavirus 2 (SARS-CoV-2) and ensure the protection of patients, physicians, and personnel without compromising patient assistance. It was recommended that elective outpatient examinations be deferred (when deemed non-essential) for reducing exposure and risk of cross-contamination, and that personal protective equipment (PPE) be rationally used.^1^^–^^3^ The use of PPE became mandatory for all personnel in the workplace, including reception workers, nursing technicians, nurses, technologists, biomedical scientists, and doctors. All patients were instructed to use masks. Specialists discussed, along with the requesting doctors, the actual need for test urgency and the most suitable cardiovascular imaging modality for each specific clinical situation. On the other hand, it is known that: (a) Cardiovascular imaging is frequently necessary for primary prevention, clinical management, and differential diagnosis in many situations, and chronically postponing it can be harmful; (b) COVID-19 can cause severe cardiovascular manifestations, especially in vulnerable individuals such as older people, immunosuppressed patients, or those with previous cardiovascular disease and/or cardiovascular risk factors (hypertension, diabetes, obesity);^4^^–^^6^(c) when the lungs are severely affected by COVID-19, the cardiac function can suffer greater impact, especially in the right ventricle. In a moment when the number of COVID-19 cases present a relative decrease, health care services will be able to progressively increase their working hours according to the pandemic tendency and guidance provided by local public authorities. Nevertheless, the reintroduction of cardiovascular imaging examinations should follow various safety protocols, as described in the next section.

## 2. Safety Protocols for the Reintroduction of Cardiovascular Imaging Examinations in the COVID-19 Era

Outpatient scheduling should be progressive and consider indications for appropriate use,^2^^,^^7^ the indication priority, risk of COVID-19, and the phase of the pandemic. Areas going through the peak transmission phase should prioritize essential examinations (high-priority cases), that is, where the results are expected to bring clinical benefits or a change in management.^1^^,^^8^ Medium-priority indications are those where examinations, although elective or in asymptomatic patients, can contribute to the implementation of primary or secondary prevention measures, in the adjustment of currently used medications, or in medium-term changes in management, potentially impacting the clinical outcome. Low-priority indications are those where tests could be deferred until after the peak of the pandemic and be gradually reintroduced once the number of cases is reduced.

Imaging examinations of patients with COVID-19, especially in outpatient settings, should be deferred (when possible) until the patient reaches the criteria for cure. At the moment of elaboration of this position statement, the Centers for Disease Control and Prevention (CDC) considers 2 criteria for releasing patients from isolation after COVID-19: The “symptom-based strategy” states that patients should be released from isolation 10 days after symptom onset; the “time-based strategy” considers that patients should be released 10 days after the first positive test (RT-PCR SARS-CoV-2).^9^ These criteria can be adjusted according to orientations of the local infection committee. If the cardiovascular examination is considered essential, it should be directed to the clinical question (focused) but sufficiently complete to avoid repetition, and follow all recommendations regarding protective measures. In addition, redundant or rarely appropriate examinations can generate an additional financial impact to that caused by the pandemic. These recommendations on indications and prioritization are valid as long as the pandemic persists and are summarized in Table 1.

**Table 1:** Indication prioritization for rescheduling cardiovascular imaging examinations according to the phase of the pandemic and COVID-19 risk

Level of priority	Prioritization rationale
**High priority** (Consider performing the examination in the next hours or the next 2 to 4 weeks)	Acute cardiovascular symptoms or with recent worsening Evaluation before urgent clinical therapy Planning of urgent cardiovascular interventions Safety monitoring of clinical therapy Monitoring after recent invasive or surgical therapy **To be performed regardless of the pandemic phase**
Medium priority	Progression monitoring of chronic myocardial disease or asymptomatic severe chronic valvular disease Clinical therapies requiring monitoring Monitoring of therapy results Initial assessment of a new and unexplained heart murmur, even if asymptomatic **Defer until the deceleration phase of the pandemic, preferably for patients at low risk for COVID-19**
Low priority	Routine evaluation of chronic disease in individuals not eligible for clinical, surgical, or invasive therapy **Defer until the control phase of the pandemic for patients at low risk for COVID-19**

*Adapted from the recommendations of the American Society of Echocardiography*.^*10*^

Defining “who,” “when,” and “how” cardiovascular imaging examinations should be used is fundamental for reducing contamination risks for the patients and health care professionals while ensuring high-quality assistance. These measures are described below.

### 2.1. Infrastructure and Safety Policies

- A triage questionnaire for defining COVID-19 risk should be applied when scheduling an appointment, at confirmation, and on the day of the examination (respiratory symptoms and contact with confirmed COVID-19 case). At admission, temperature checks should also be performed. The same triage protocol should be applied to the staff.
- Instructions regarding social distancing, mask use, and hand hygiene should be provided when scheduling an appointment and reinforced at admission.
- Telemedicine applications should be implemented.
- Visual displays containing information on protective measures should be placed on waiting rooms and strategic places (in more than one language).
- Hand sanitizer should be widely available.
- Patients should be instructed to arrive on time or to wait for their turn inside the car; the number of accompanying persons should be limited.
- The number of seats in the waiting room should be reduced so as to guarantee social distancing.
- Acrylic glass barriers or distancing cones should separate patients and the reception staff.
- The time between examinations should be extended for avoiding crowded rooms and allowing proper cleaning. Facilities should consider opening at non-business hours or weekends.
- Equipment and surface cleaning protocols should be performed after each examination according to the local infection control policies and the type of examination.
- Communication with patients or financial transactions should be preferably performed online.
- There should be 2 patient flows: One for patients with suspected or confirmed COVID-19 and another for patients without the disease. Rooms, equipment, and circulation areas should be separated for the 2 groups.
- Ideally, rooms should be well-ventilated and have negative pressure when performing procedures that generate aerosols (transesophageal echocardiography [TEE], exercise stress tests).
- PPE stocks should be continuously monitored and maintained.
- Contaminated material should be disposed of according to health surveillance agency guidelines.

### 2.2. Prioritizing Indications and Choosing the Cardiovascular Imaging Method

- Define the priority level of the examination (according to the local phase of the pandemic and the risk of COVID-19 [Table 1]).
- Select the best test for providing essential information on the patient's clinical condition.
- Consider replacing an examination for another with similar accuracy, but lower risk of COVID-19.
- Avoid performing multiple tests or inappropriately repeating the same examination.

### 2.3. Personnel Protection

- Practice frequent hand hygiene and constant mask use.
- Appropriately use PPE according to the required protection level (for droplets or aerosols). Table 2 summarizes the use of PPE according to the protection level required by each type of examination, the risk of COVID-19, and location.
- Participate in frequent institutional training on donning and doffing PPE, its duration of use and storage, as well as hand hygiene. Evidence shows that the highest chances of infection in professional settings occur at the moment of inadequate PPE removal. Steps and guidance for donning and doffing PPE are demonstrated in Figures 1 and 2.
- Limit the number of professionals in the examination and report rooms.
- In case of examinations that require close contact between the professional and the patient, such as echocardiography and vascular ultrasonography, consider the use of a plastic or acrylic glass barrier.
- Limit test duration by using more objective or focused protocols. In case of patients admitted with suspected or active COVID-19, ultrasound/echocardiography examinations should be performed at the bedside, without electrocardiographic monitoring. Images should be obtained first and measurements should be performed only after the professional leaves the room and the equipment is disinfected.
- If possible, concentrate appointments of patients with suspected or confirmed COVID-19 within a specific time frame for minimizing exposure and rationalizing the use of PPE.

**Table 2:** Use of personal protective equipment during cardiovascular imaging examinations in the COVID-19 era according to exposure risks

	TTE, CCT/CMR, pharmacological stress test (Echo/SPECT/PET/CMR)	TEE, exercise stress test (Echo/SPECT/ scintigraphy)
Low risk for COVID-19	Standard safety level Mandatory hand hygiene Surgical mask	Protection against aerosols:** Mandatory hand hygiene N-95/PFF2 respirator* Isolation gown (preferably waterproof) Examination gloves Surgical cap Face shield; protection goggles when face shields not available Ideally, place patients with COVID-19 in negative pressure rooms Attempt to replace examination for an alternative method
Suspected or confirmed COVID-19 cases	Protection against droplets Mandatory hand hygiene N-95/PFF2 respirator* Isolation gown Head covering Examination gloves Safety goggles	

* *In case of complete unavailability of N-95 respirators or equivalents, wear a single-use surgical mask in addition to a face shield*.

** *Also applicable to all examinations performed with patients admitted in intensive care units (ICUs), on invasive or non-invasive ventilation. These recommendations should be followed by all personnel directly involved in the procedure (physicians, nursing technicians, technologists, etc.). CCT: cardiovascular computed tomography; CMR: cardiovascular magnetic resonance imaging; Echo: echocardiography; PET: positron emission tomography; SPECT: single-photon emission computed tomography; TEE: transesophageal echocardiography; TTE: transthoracic echocardiography*.

**Figure 1:** Sequence for donning personal protective equipment.

**Figure 2:** Sequence for doffing personal protective equipment.

### 2.4. Equipment Maintenance

- Restrict the PPE components to those minimally necessary for performing the examination in order to reduce the need for cleaning and disinfection after the procedure, as well as the risk of contamination and cross-transmission.
- Consider the use of a protective cover for the equipment and transducer (in case of ultrasound equipment), as long as it does not hinder equipment use or prolong test duration.
- All equipment and accessories should be cleaned and disinfected after each use according to equipment disinfection guidelines. Stress tests and TEE, or examinations performed on patients with aerosol emission (in the intensive care unit [ICU], on invasive or non-invasive ventilation) require longer appointments, since disinfection should be prolonged. Cleaning and disinfection protocols are detailed on Supplementary Material.

### 2.5. Special Precautions for Stress Tests

Stress tests are essential in the evaluation of patients with suspected or confirmed coronary heart disease. They include exercise or pharmacological stress tests with any of the nuclear cardiology imaging modalities (single-photon emission computed tomography/positron emission tomography [SPECT/PET]), echocardiography, or cardiovascular magnetic resonance imaging (CMR). However, stress tests can increase the risk of contamination by droplets and should be deferred (for patients at low risk for COVID-19) or cancelled (for patients with suspected or confirmed COVID-19). Pharmacological stress should have preference over exercise. Once the pandemic fades, when clinically appropriate, exercise stress tests should include additional precautions, such as:^1^

- Assess the facility's air circulation patterns – consult with the engineering department on optimized equipment/staff. Given the uncertainty regarding the aerosol generating capacity of a stress test, an exclusive room should be used for exercise tests, if possible, with negative pressure.
- Avoid manual blood pressure measurement, if possible. Automatic blood pressure measurement is commonly used and reasonably precise both in stationary patients or those subjected to pharmacological stress.
- The supervising team should keep a distance (2 meters) from the patient, when possible.
- All staff involved in the test should wear a face shield (particularly during exercise stress echocardiography) and gloves, in addition to the PPE required for all examinations.
- When possible, the patient should be encouraged to wear a mask while exercising.
- If exercise is deemed necessary, consider testing for COVID-19 before the test.
- A careful choice of exercise protocol should be made since longer exercises increase the duration of patient-team interaction. The bicycle protocol is associated to lower levels of peak minute ventilation.

### 2.6. Special Precautions for TEE

Among all the echocardiography modalities, TEE is probably the one with the highest risk of health care team contamination due to the handling of the patient's airways, contact with fluids, close contact between the patient's mouth and the professional, and to the cough reflex that can happen during the probe passage towards the esophagus. Indications for this test should thus be carefully evaluated, and the highest precaution level is recommended (even for tests performed in the operating room or in patients that do not have suspected or confirmed COVID-19)^2^^,^^3^^,^^8^^,^^12^^,^^13^ (see Table 2). Ideally, TEE should be performed in an exclusive room containing protective covers for the echocardiography equipment (isolation or waterproof covering) and for all the necessary equipment, in addition to a strict and lengthy disinfection protocol for environmental surfaces between each test (approximately 1 hour).

### 2.7. Special Precautions for Pediatric Echocardiography

Considering that children have a higher possibility of being infected with COVID-19 and be asymptomatic or show minimum symptoms, the triage measures that apply to adult patients can be insufficient, requiring adjustments in outpatient or inpatient echocardiography. In case of an active and cooperative patient, the echocardiography physician should preferably be the only person in contact with the child. Children under 2 years old have difficulties wearing masks, which leads to a higher risk of exposure to the virus. In addition to the main form of transmission of SARS-CoV-2 (through respiratory droplets), transmission through fomites has been assumed plausible.^14^ Therefore, diaper changes should be avoided, if possible, during the examination and if necessary, performed with adequate hygiene. Due to the higher risk of an asymptomatic form of COVID-19 in children, some facilities located in endemic regions choose to test new pediatric hospital admissions for SARS-CoV-2.

### 2.8. Special Precautions for Fetal Echocardiography

In facilities where fetal echocardiography (FE) is performed within the cardiology sector, pregnant women should be separated from pediatric patients, both in waiting and procedure rooms. Differently from previous viral outbreaks (H1N1, SARS-CoV, MERS-CoV), which were associated to severe complications in pregnant women, current information (although limited) suggests that these patients are not more susceptible to SARS-CoV-2 infections or, if infected, are not more prone to developing severe complications.^15^ However, given the uncertainty and possibility of an increase in risk as new data become available, the CDC alerts that pregnant women should protect themselves. Up to one accompanying person is allowed; for reducing exposure, the echocardiography room should ideally be occupied only by the pregnant patient and the examining physician.

## 3. Echocardiography in Adult Patients in the COVID-19 Era

Echocardiography is the first-line method in the diagnosis, prognostic evaluation, and therapeutic guidance of various cardiovascular diseases. During the new coronavirus outbreak, it remains a crucial imaging method, mainly due to its portability in comparison to other methods; this allows bedside examinations for isolated and/or critically ill patients.^13^ However, since it entails a close contact between the physician and the patient, echocardiography poses a high risk of infection by COVID-19.^10^

### 3.1. Prioritization and Indications for Transthoracic Echocardiography in Adult Patients at Low Risk for COVID-19

Even in patients at low risk for COVID-19, the reintroduction of outpatient examinations should be progressive, considering priority criteria and the phase of the pandemic (see Table 1).^10^^,^^11^^,^^13^ Next, we describe indications for each priority level.

#### 3.1.1. High-priority Examinations (Essential Procedures)

- Individuals with acute cardiovascular symptoms or with recent worsening. Examples: New York Heart Association (NYHA) class III or IV heart failure, probable cardiac syncope, chest pain, arrhythmias, stroke, suspected acute valvular heart disease (mitral or aortic regurgitation), acute symptoms in patients with a prosthetic heart valve, or suspected symptomatic severe aortic stenosis with no previous diagnosis.^7^
- Evaluations before urgent clinical therapy, even in asymptomatic patients. Examples: baseline echocardiography prior to the initiation of chemotherapy or evaluation of left ventricle ejection fraction (LVEF) prior to implantation of a cardioverter defibrillator for primary prevention.
- Planning of urgent cardiovascular intervention: mitral valve repair, transcatheter aortic valve replacement (TAVR), left atrial appendage occlusion.
- Safety monitoring of clinical therapy. Example: chemotherapy follow-up in patients at high risk for cardiotoxicity, even if asymptomatic.
- Follow-up after recent invasive or surgical therapy. Example: suspected pericardial effusion after device implantation or heart surgery, even if asymptomatic.
- Suspected infective endocarditis with a high pre-test probability.
- Suspected pericardial disease or progression of pericardial effusion.

Indications for urgent (or high-priority) echocardiography in hospitalized patients are generally the same as before the pandemic (mechanical complications after acute myocardial infarction, tamponade, aortic dissection, and others).

#### 3.1.2. Medium-priority Examinations

- Monitoring of the progression of chronic myocardial disease or asymptomatic severe chronic valvular disease. Examples: cardiomyopathy, aortic stenosis, primary mitral regurgitation, prosthetic valve dysfunction.
- New symptoms in patients with known heart or lung disease.
- Heart failure with reduced LVEF (HFrEF), when LVEF requires medical therapy or device implantation.
- Assessment before a routine procedure or therapy. Example: non-urgent surgery.
- Monitoring of therapy results. Example: treatment of regressing dilated cardiomyopathy or heart transplant rejection, Takotsubo syndrome (stress cardiomyopathy), Kawasaki disease, right ventricular (RV) dysfunction after pulmonary embolism (PE), pericardiocentesis, assessment of ventricular assistance device.
- Initial assessment of new unexplained heart murmur.

These examinations can be reintroduced in areas where the pandemic is waning.

#### 3.1.3. Low-priority Examinations

These are elective examinations, requested annually or every two years for the follow-up of patients with asymptomatic chronic diseases or who have not shown changes in their health state, where the test result will not change treatment and/or short-term outcome. These examinations can be deferred to when virus transmission is reduced or restrictions are suspended or made flexible, especially if the patient has been subjected to echocardiography in the last 12 months.^16^

### 3.2. Prioritization and Indications for Transthoracic Echocardiography in Adult Patients with Suspected or Confirmed COVID-19

From a cardiovascular point of view, patients affected by SARS-CoV-2 can display evidence of myocardial dysfunction (both left and right), vascular alterations, arrhythmias, thromboembolic phenomena, and pericardial effusion.^4^

Therefore, echocardiography can also contribute to the clinical assessment of patients with COVID-19, in the following situations:^2^^,^^3^^,^^17^^–^^20^

- Incapacitating dyspnea. Dyspnea is very common in patients with pneumonia secondary to COVID-19 (a situation where troponine levels are also increased, possibly leading to a false hypothesis of myocarditis). In this case, normal levels of B natriuretic peptide (BNP), even with elevated troponine, can exclude the need for echocardiography. Lung ultrasonography, in experienced hands, can help in the differential diagnosis between heart failure and pneumonia.
- Patients with previous heart disease with changes in hemodynamic state or signs and symptoms of disproportionate involvement of the lungs.
- Cardiomegaly on chest X-ray.
- Clinically significant arrhythmias or those with abrupt beginnings.
- Chest pain with electrocardiographic alterations and/or elevated troponin levels. In case of strong myocarditis suspicion and if CMR is indicated as crucial for the treatment, echocardiography can be initially omitted.
- Hemodynamic instability, respiratory failure, and/or shock of uncertain etiology.
- Suspected pulmonary hypertension and/or right ventricular dysfunction.

In difficult acoustic windows, the use of an echocardiographic contrast agent can be employed to ensure test results, reduce test duration, and avoid inadequate diagnosis or other unnecessary tests.^21^^,^^22^

In patients with COVID-19, the examination should be focused on the clinical question. Serial echocardiography should be avoided unless there is clear change in the clinical state (hemodynamic instability). However, in the ICU, the echocardiogram is frequently used for monitoring the progression of critically ill patients, especially regarding fluid management. In this case, a focused ultrasound or focused echocardiography can be used. Protocols for these cases are described below.

### 3.3. Protocols for Transthoracic Echocardiography in Adult Patients in the COVID-19 Era

#### 3.3.1. Focused Echocardiography

This examination is focused on the clinical question, but is sufficiently complete for including all clinical hypotheses. The protocol should be performed by echocardiographers in individuals with COVID-19 without electrocardiographic monitoring, and include the assessment of the following parameters:^2^

- Left ventricle: quantitative assessment of global systolic function (ejection fraction), signs of regional myocardial dysfunction, and cavity size.
- Right ventricle: assessment of global function, fractional area change (FAC) or tricuspid annular plane systolic excursion (TAPSE), cavity size, and tricuspid regurgitation velocity and pressure gradient, if possible.
- Valves: basic anatomical assessment and color Doppler; in case of signs of dysfunction, evaluate further.
- Pericardium: assessment of thickening or effusion.

#### 3.3.2. Focused Ultrasound – Indications, Protocols, and Main Findings

The focused ultrasound, also known as point-of-care cardiac ultrasound, is widely used as a support tool in the diagnosis, management, and monitoring of patients with COVID-19.^23^ The biggest advantages of this technique in patients with COVID-19 include its general availability in urgency/emergency settings and ICUs, its high diagnostic accuracy, and the wide range of clinical information that can be obtained through this examination. The focused ultrasound is also easily performed as a bedside examination – avoiding patient transportation to the radiology service and virus spread within the hospital. This test can be performed both with conventional ultrasound equipment or with portable or ultraportable (pocket-sized) devices; these are preferred due to easier disinfection and bedside use. All personal and equipment protective measures previously described should be performed. Among the applications of focused ultrasound in patients with COVID-19, we highlight lung and cardiac ultrasonography.

#### 3.3.3. Focused Lung and Pleural Ultrasonography

Lung ultrasound (LUS) represents a fast alternative for evaluating the degree of pulmonary involvement and monitor the result of therapeutic interventions at the bedside. The diagnostic accuracy of LUS is similar to that of chest computed tomography (CT) in patients with respiratory complaints such as dyspnea and hypoxemia (85% sensitivity, 93% specificity for non-COVID-19 pneumonia).^24^ Normal LUS findings have also presented an excellent correlation with chest CT results with no alterations of lung parenchyma such as ground-glass opacities. Therefore, LUS presents a high negative predictive value, allowing its use in patient risk stratification.^25^ In addition to diagnosis and initial risk stratification, LUS is being widely used in the monitoring of critically ill patients COVID-19.^26^ It contributes to decision-making on prone ventilation, extracorporeal membrane oxygenation (ECMO), and weaning from mechanical ventilation in acute respiratory failure.^27^ LUS is also useful for ruling out other pathologies such as pneumothorax, which can occur in individuals on positive pressure ventilation.

Various LUS protocols are described in the literature. Regarding patients with COVID-19, at least 6 regions should be assessed in each hemithorax: anterior, anterior axillary and posterior axillary regions above and below a line at the fourth intercostal space. Since COVID-19 lung affections are bilateral, multifocal, and not necessarily uniform, we highlight the importance of not focusing on specific areas of the thoracic wall and recommend a full chest assessment.^28^^,^^29^ LUS findings in patients with COVID-19 are able to monitor the progression and extension of pulmonary affections identified by the chest CT and are described in Figure 3. Notably, pleural effusion is not a common finding of COVID-19, hence it indicates a low probability of SARS-CoV-2 infection. Throughout the recovery period, the lungs gradually return to normal; ultrasound assessment indicates that subpleural consolidations disappear, the pleural line normalizes, and A-lines reappear.^30^

**Figure 3:** Lung ultrasound findings in patients with COVID-19 and their correlation with disease severity and tomography findings

#### 3.3.4. Focused Cardiovascular Ultrasound

Focused cardiovascular ultrasound is being used by emergency physicians or intensivists for rapid assessment and screening for pre-existing cardiac and vascular diseases, as well as in the early detection of COVID-19-related myocardial alterations.^23^ The objective of this ultrasound is to quantitatively assess left ventricular systolic function, right ventricular size and contractility, inferior vena cava size and collapsibility, in addition to severe vavular abnormalities and pericardial effusion.29 (Figure 4). A brief screening of patients with COVID-19 is indicated in the presence of high troponin and BNP in addition to electrocardiographic or hemodynamic changes, or in the case of suspected PE.31 In this case, one of the main advantages of focused ultrasound is that it reduces the need for conventional echocardiography, thus also decreasing the physicians’ exposure and the need for equipment decontamination while also saving PPE. This examination is not equivalent to echocardiography, but it is capable of confirming or excluding a specific diagnosis during a quick bedside evaluation, thus contributing to therapeutic decisions. The focused ultrasound can also be used in the triage of patients that need echocardiography. On the other hand, without proper training, there is a risk of acquiring inadequate images that lead to false positive or false negative diagnoses. This could in turn result in unnecessary treatments that can be harmful, in inappropriate echocardiography indications, or in treatment delays. Therefore, the use of focused cardiac ultrasound should be based on institutional protocols, and we recommend an internal competence-based certification as well as constant quality assessments, considering that teaching and training methodologies for this method vary greatly.

**Figure 4:** Protocols for the focused cardiovascular ultrasound. Windows, main findings, and possible diseases.

Integrated (lung and cardiac) focused ultrasound is ideal for adequate characterization of volume status, subjacent ventricular function, monitoring of fluid state, and titration of vasopressor drugs in case of inotropic support. In patients with RV dysfunction, focused vascular ultrasound for investigating deep vein thrombosis (DVT) can complete the assessment.

### 3.4. Prioritization and Indications for Stress Echocardiography in the COVID-19 Era

In patients at low risk for COVID-19, with proper indication and when deferring is not possible or recommended (such as in preoperative cancer patients or those with a high pre-test probability of obstructive coronary artery disease [CAD]), pharmacological stress tests are preferred because they are not considered aerosol-generating. Another alternative for investigating selected cases of chronic coronary arterial disease during the pandemic is to prioritize computed tomography (CT) coronary angiography.^10^ Once a very low local prevalence of COVID-19 is reached, exercise echocardiography can return to being the first choice, but with additional safety procedures as previously described.

### 3.5. Prioritization and Indications for Transesophageal Echocardiography in Adult Patients in the COVID-19 Era

TEE causes additional concern, since the risk of equipment and professional contamination through droplets and aerosols is very high. Therefore, the incremental value of TEE in relation to transthoracic echocardiography (TTE) should be carefully evaluated on a case-by-case basis together with the assistant physician. For the duration of the pandemic, alternative methods should preferably be chosen, especially in patients with COVID-19.^2^^,^^12^ One of the main indications for TEE — screening for intracavitary thrombi — can be performed through CT angiography, which presents a lower risk of staff contamination. This alternative is obviously only available for patients who can be transported and with preserved renal function. Whenever possible, complete diagnosis should be achieved through TTE, leaving TEE for critical situations with possible changes in management, especially in the ICU or operation room where no other methods are available and the clinical question should be promptly answered. In areas where the pandemic is decreasing, the reintroduction of TEE for outpatients should follow strict criteria, and each case should require an individual evaluation. It is recommended that outpatient scheduling be restored based on patient priority, considering one's previous position on the waiting list, clinical indications and conditions (symptomatic or asymptomatic), and potential impact of the examination in the patient's clinical history (for example, tests required for further procedure scheduling).^2^^,^^12^^,^^13^ Additional measures for TEE scheduling and test safety are described in Table 2. Among the various indications for TEE, high priority is given to the following:^32^

- Infective endocarditis with valvular or paravalvular involvement.
- Type A aortic dissection (Stanford) in unstable patients (if stable, prefer CT; if associated with suspected aortic insufficiency, perform TTE).
- Beginning of mechanical circulatory support.
- Acute myocardial infarction with suspected mechanical complications not detected by TTE (ventricular septal defect, left ventricular free wall rupture, or papillary muscle rupture).
- Prosthetic valve dysfunction poorly defined in TTE or CT.
- Monitoring of patients on venovenous ECMO for treating COVID-19 pneumonia.
- Intraoperative evaluation of the result of mitral valve repair, septal myectomy, or in the diagnostic and management of complications.
- Hemodynamic instability due to undifferentiated shock in individuals with inadequate acoustic windows for TTE (such as those in the perioperative period of cardiac surgery or on prone ventilation).
- Screening for thrombi in the left atrial appendage prior to electrical cardioversion for restoring sinus rhythm in an unstable patient or with unavailable cardiovascular computed tomography (CCT) (CCT with low contrast infusion is the first choice for patients with COVID-19).

TEE is not recommended in the following cases:

- Screening for infective endocarditis in patients with transient fever and no bacteremia or new heart murmur.
- Transient bacteremia with identified pathogen not typically associated to infective endocarditis or with documented non-endovascular source of infection.
- Re-evaluation of a previous echocardiogram in stable individuals when no changes in therapy are being considered.

## 4. Pediatric, Congenital, and Fetal Echocardiography in the COVID-19 Era

The echocardiographic examination in children can result in increased risk for the staff and community, considering that although these patients have a reduced occurrence of severe COVID-19, many infected children can be asymptomatic or minimally symptomatic. In addition, pediatric care requires the company of an adult, thus demanding adjustments in specialized care. Since the pediatric population with congenital heart defects is different from the corresponding adult one regarding the risk of COVID-19 transmission and indications for echocardiography, the aim of this topic is to update indications and protocols for TTE and FE in this population.

### 4.1. Prioritization and Indications for Transthoracic Echocardiography in Pediatric and Congenital Patients

Within the pediatric age group and considering patients with congenital heart defects, absolute indications include suspected congenital heart defect, postoperative and preoperative evaluation of congenital heart defects, children with acquired heart disease, heart transplant, increased risk of impaired cardiac function (chemotherapy treatment), and cardiac complications associated with respiratory infections.^33^^,^^34^ These indications should be categorized as high, medium, or low priority, as demonstrated in Table 2, when reintroducing examinations at the outpatient level according to the hemodynamic repercussions and clinical reasoning.

### 4.2. Optimization of the Transthoracic Echocardiography Protocol in Pediatric and Congenital Patients

TTE should be performed in the usual manner due to the wide anatomical variability and challenges regarding the evaluation of systolic and diastolic function, thus leaving focused assessment for other applications such as the emergency sector, pediatric intensive care, and in suspected of confirmed COVID-19 patients. In case of a potentially complicated examination, the patient should be referred to a more experienced physician; this increases the probability of an adequately detailed, precise, and quick examination without the need for additional practical support. Recently, an alert regarding multisystem inflammatory syndrome in children and teenagers has been released; this disorder has been described as a generally late clinical presentation associated with COVID-19, characterized by similar manifestations to those of the typical or incomplete Kawasaki syndrome and/or septic shock syndrome.^35^ The most affected age range includes school-age children (mean age 9 years), which differentiates this disorder from the Kawasaki syndrome; symptoms are predominantly gastrointestinal. Virtually 100% of cases have heart affections with LVEF impairment, which has been described by various groups. In addition, patients present cardiogenic shock and coronary artery involvement with variable degrees of dilation, and some groups have reported aneurysms. Respiratory symptoms are mild, and skin rash and mucosal involvement may also occur.^35^^,^^36^ In case of clinical suspicion of multisystem inflammatory syndrome in children and teenagers, the main objectives of echocardiography are to identify possible left ventricular dilation, measure systolic function through LVEF, quantify the valvular regurgitation, evaluate the morphological aspect of coronary arteries (dilation and/or aneurysm), and pericardium.

### 4.3. Prioritization and Indications for Fetal Echocardiography

FE assessment faces challenges in the final stage of pregnancy where perinatal and neonatal planning and decision-making are required. At first, patients with low-risk pregnancies were instructed to not perform FE; those with moderate-risk pregnancies should defer FE for when the COVID-19 risk was reduced or until 28 weeks of pregnancy; finally, women with high-risk pregnancies should promptly schedule and perform the examination.^15^However, as the pandemic progressed, considering that pregnancies would continue and fetal cardiac diseases can be critical, we recommend that FE be performed according to the previously published guidelines.^37^^,^^38^ The examination should be performed following PPE protocols for the pregnant patient and health care professional. Echocardiographic assessment should be as complete as possible, avoiding re-evaluations.

The possibility of prenatal or perinatal infection should be considered when newborns are transferred to the pediatric or cardiac neonatal intensive care unit after birth. Data on the vertical transmission of SARS-CoV-2 are still scarce;^39^^,^^40^ however, if the new mother tests positive for the virus in the 14 days after birth, the newborn should also be tested and managed as a positive case until a negative result is confirmed.

Echocardiographic tests in the pediatric population and in patients with congenital heart defects remain crucial during the SARS-CoV-2 pandemic and their health care should be guaranteed, minimizing risks for the professional team, patients, and the general public.

### 4.4. Prioritization and Indications for Transesophageal Echocardiography in Pediatric Patients

In patients with congenital heart defects, TEE is considered a part of intraoperative care and hemodynamic interventions. In outpatient cases, considering the high risk of exposure to SARS-CoV-2, this examination should be deferred or substituted for an alternative modality such as TTE associated with agitated saline contrast, cardiovascular computed tomography (CCT) or CMR with contrast agents. As for children, the risks and benefits of aerosol-emitting procedures should be weighed along with those of patient transportation, disinfection of the CCT/CMR room, contrast administration or radiation in CCT, in addition to prolonged exposure in case of CMR.

### 4.5. Protocol for Transesophageal Echocardiography in Pediatric Patients

Due to the unreliability of using symptoms in the prediction of COVID-19 status in children, a specific recommendation has been proposed for TEE in these patients:^15^

1. All pediatric patients should be considered positive for COVID-19 in TEE, unless with negative tests in the last 48 to 72 hours. In case of a negative test, TEE can be performed using standard precautions (gloves, mask, and eye protection).
2. In pediatric patients without negative COVID-19 tests in the last 72 hours, who were intubated before arriving to the operation room, the risk of producing aerosols is considered low. The probe can be introduced by the anesthesiologist or examining physician, according to the institution's standard procedures and precautions.
3. In asymptomatic patients without negative COVID-19 tests in the last 72 hours who require intubation in the operation room, this procedure should be performed by the anesthesiologist using appropriate PPE and air purifying respirators. This process should be followed by a 20- to 30-minute waiting period, depending on local protocols and environmental factors, for allowing complete room air exchange. During this period, people should not be allowed in the room. The TEE probe should ideally be introduced by the anesthesiologist immediately after airway stabilization for minimizing the exposure of other professionals. After the waiting period, the examining physician can manipulate the probe according to standardized procedures and precautions.
4. Children with positive COVID-19 tests or with symptoms should be isolated. Probe introduction by the anesthesiologist is strongly advised for minimizing the exposure of other professionals. The team of health care professionals in the operation, recovery, or procedure rooms should use strict isolation equipment at all times and be trained in PPE donning and doffing. Only essential personnel are allowed in the operation room (only one echocardiography physician) for reducing exposure risk and saving PPE.

## 5. Vascular Ultrasonography in the COVID-19 Era

One of the situations requiring the biggest caution in patients infected by the SARS-CoV-2 virus is the development of coagulopathies, mostly characterized as a tendency for venous, arterial, and microvenous thrombosis. Klok et al.^31^ evaluated the incidence of venous thromboembolism (VTE) and arterial thrombotic complications in 184 patients with COVID-19 admitted to an ICU. Despite the VTE prophylaxis performed in all patients, a 31% incidence of thrombotic complications was observed.^31^ Accordingly, there was an increase in demand for vascular ultrasonography (VUS) during the pandemic, especially for the venous VUS in patients with COVID-19, most of which were hospitalized. For this reason, a deeper discussion on this examination is performed below.

### 5.1. Prioritization and Indications for Vascular Ultrasonography in Patients at Low Risk for COVID-19

Considering outpatients without COVID-19, the types of examinations should be categorized according to indication priority (see Table 1). High-priority (essential) examinations include venous VUS for DVT screening, as well as the carotid and vertebral artery VUS in patients with suspected stroke. Considering patients with peripheral obstructive disease, a priority for arterial VUS should depend on surgical treatment indications.

Other examinations should be categorized as medium or low priority by the assistant physician, as long as there are no indications for urgent invasive treatments. These include VUS of the aorta and its branches, VUS for varicose vein assessment, and carotid artery VUS in screening for carotid artery disease in the preoperative period of cardiac surgery.

### 5.2. Prioritization and Indications of Vascular Ultrasonography in Patients with COVID-19

- Suspected pulmonary thromboembolism (PTE): VUS has a low accuracy in diagnosing PTE, but can be indicated in case of high bleeding risk, when the result should change management, or when PTE suspicion is high and there is no available CT angiography.^41^
- Suspected DVT: In individuals with high clinical suspicion of DVT and bleeding risk, lower limb venous VUS is recommended.^41^
- Suspected acute arterial occlusion of upper or lower limb (arterial VUS).
- Stroke of unknown cause (carotid and vertebral artery VUS).

### 5.3. Situations Where Vascular Ultrasonography is Not Recommended for Patients with COVID-19

Venous VUS should not be used as a marker for altering a patient's anticoagulant therapy management.

Laboratory tests cannot indicate the need for this examination; hence, high D-dimer values do not justify VUS for DVT screening. In case of negative D-dimer results, there is no need for VUS in DVT screening.

Upper limb DVT has low morbidity in critically ill patients, thus there is no routine recommendation for venous VUS in this region.

In conclusion, VUS is not recommended in situations where its results will not determine changes in management or are not a prerequisite for urgent surgery.

### 5.4. Optimization of Vascular Ultrasonography Protocols

The complete venous VUS protocol is the recommended method for investigating DVT; however, point-of-care 3- or 2-point compression ultrasound in critically ill inpatients with COVID-19 seems to be a reasonable option, except if the pain reported by the patient is located in the infrapatellar segment (then, the complete protocol should be performed). The 3-point protocol assesses compressibility in all proximal veins of the evaluated lower limb. The 2-point protocol evaluates the compressibility of the common femoral vein 1 to 2 cm above and below the saphenofemoral junction (in the inguinal fold) and in the popliteal vein up to the confluence of the leg veins.^42^ Total or partial absence of compressibility in the affected vein, as well as vascular swelling due to an intraluminal thrombus detected by 2-dimensional techniques, are ultrasonographic signs of DVT. The sensitivity of the 3-point compression method is considered greater than that of the 2-point method (90.57% vs 82.76%), with similar specificity (98.52%).^43^

In addition to a shorter exposure period for the professional, the point-of-care VUS protocol can be performed by the emergency physician, provided proper training is involved. After the pandemic, when possible, the complete protocol should be given preference.

Other VUS examinations, such as the carotid and vertebral artery VUS and limb arterial VUS, when necessary, should follow previously established protocols.^44^ All protective and PPE measures should be followed as previously discussed.

## 6. Cardiac Magnetic Resonance in the COVID-19 Era

### 6.1. Prioritization and Indications

The COVID-19 pandemic caused an important reduction in the number of outpatient requests for CMR, leaving only high-priority examinations: Clinical suspicion of myocarditis, differential diagnosis of cardiac masses, as well as other exceptional situations such as the assessment of complex ventricular arrhythmias.

CMR is well-defined as the preferred method for the reliable assessment of ventricular function, cardiac volumes, ischemia, myocardial viability, as well as in the detection of areas of myocardial fibrosis, study of infiltrative and deposition diseases, structural evaluation of patients with cardiac arrhythmias, and in specific cases, for complementing the echocardiographic evaluation of valvular and congenital heart diseases. In patients with low risk for COVID-19, prioritization should follow the rationale presented in Table 1. CMR has the advantage of providing, with one test, a global assessment of the heart with multiple functional and structural data, thus avoiding multiple trips to the hospital or clinic; this reduces patient circulation and optimizes resources.^45^In patients with COVID-19, CMR allows the diagnostic of myocarditis, perimyocarditis, acute myocardial infarction with no evidence of obstructive coronary artery disease (MINOCA), Takotsubo syndrome, and the differentiation between ischemic and inflammatory presentations. Conversely, the patient's risk of transmission and instability in the acute phase should be carefully evaluated and the examination could be deferred, whenever possible, until the criteria for cure are attained. On the other hand, a recent study evaluated myocardial injury in patients who had recovered from COVID-19 and showed a high prevalence of nonischemic delayed myocardial enhancement and preserved systolic function, already with no evidence of edema, suggesting permanent myocardial damage.^46^ CMR can thus be an important tool for better comprehending the mechanisms of myocardial injury and for assessing myocardial damage after recovery from COVID-19.

### 6.2. Optimizing Cardiac Magnetic Resonance Protocols

CMR protocols should be reduced (maximum 30 minutes) for all clinical indications; the focus should be on evaluating myocardial function through cine magnetic resonance imaging (MRI), while myocardial tissue characterization should be performed through late gadolinium enhancement. A T2 weighted anatomical sequence can be performed for detecting myocardial edema in case of suspected acute myocardial inflammation. When available, T1, T2, and/or T2* mapping associated with cine MRI sequences and late enhancement constitute an optimized and efficient protocol.^11^ When evaluating myocardial ischemia, the physician should focus on perfusion sequences under pharmacological stress, subsequently adding cine and late enhancement sequences after stress. CMR can also be employed in the diagnosis of intracavitary thrombi, with a fast and directed study using late gadolinium enhancement and avoiding the need for TEE, thus reducing the examining physician's exposure to potential contamination. The evaluation of cardiac masses is adequately performed with high sensibility using CMR and can identify benign or malignant characteristics; it is though quite limited in the diagnostic of vegetations due to their small dimensions and mobile characteristics. Mass characterization should follow the routine protocol, which uses cine MRI sequences, T1 weighted anatomical sequences with or without fat suppression, T2, rest perfusion, and late enhancement, always focused on mass localization. Congenital diseases can have optimized assessment with the use of 3D resonance angiography associated with cine MRI sequences, and in case valve assessment is needed for complementing the echocardiography, ventricular function assessment through cine MRI should be prioritized and protocols should be directed to the valvular apparatus with flow mapping sequences.^47^

## 7. Cardiovascular Computed Tomography in the COVID-19 Era

### 7.1. Prioritization and Indications

CCT can be used in the evaluation of multiple forms of heart diseases in all stages of the COVID-19 pandemic in a fast, efficient, and safe manner.^48^ For this, depending on the local stage of the pandemic, the risk of exposure to the virus in the examination should be weighed with the benefit of its results for patient management and treatment.^11^ Table 3 proposes the prioritization of indications during the COVID-19 pandemic.

**Table 3:** Prioritization of CCT indications during the COVID-19 pandemic

	Low-priority indications	Medium-priority indications	High-priority indications (consider performing in the next hours or in up to 2 to 4 weeks)
CAD	Calcium score in asymptomatic patients		Acute chest pain with high enough suspicion of CAD
	Stable chest pain without high suspicion of CAD		Stable chest pain with high risk of cardiovascular events, or when high-risk anatomy is suspected	
Structural cardiac disease	Patients with stable structural cardiac disease (TAVR, TMVR, LAA occlusion, together with the Heart Team)		Patients requiring urgent structural intervention (TAVI, TMVR, LAA occlusion)
Atrial fibrillation	Assessment of pulmonary veins for planning atrial fibrillation ablation	Evaluation of the left atrial appendage in **chronic** atrial arrhythmia before restoring sinus rhythm	Evaluation of the left atrial appendage in **acute** atrial arrhythmia before restoring sinus rhythm
Heart failure	Investigation of CAD as cause of heart failure in patients with stable cardiomyopathy		Hospitalized patient with acute cardiomyopathy and low or intermediate probability of CAD, where CCT can change management
			Evaluation of left ventricular assist device malfunction
Valvular	Assessment of aortic stenosis severity	Subacute or chronic prosthetic valve dysfunction	Acute and symptomatic prosthetic valve dysfunction, endocarditis, perivalvular extension of endocarditis or possible abscess
Masses/ congenital	Suspected benign cardiac masses or those unlikely to require biopsy or surgical planning		New suspected malignant cardiac masses or those requiring biopsy or surgical planning
	Elective assessment of congenital anomaly		Ruling out left ventricle thrombus after inconclusive echocardiography, when alternative methods (magnetic resonance) are not available

*CAD: coronary artery disease; TAVR: transcatheter aortic valve replacement; TMVR: transcatheter mitral valve replacement; LAA: left atrial appendage; CCT: cardiovascular computed tomography.*

As for indications, the main diagnostic and prognostic characteristics of CCT should be taken into consideration when making a decision:^49^

- The ability of CCT to precisely exclude high-risk CAD can prevent admissions and save resources, as well as recommend hospitalization for patients with detected disease (especially those reluctant to seeking emergency care).
- The fundamental role of CCT in the preoperative anatomical assessment of structural cardiac diseases reduces the risk of acute and chronic complications associated with interventions.
- CCT can be preferred over TEE for ruling out thrombi in the left atrial appendage and intracavitary thrombi before cardioversion, reducing chances of cough and aerosol generation related to TEE.
- In patients suspected or confirmed for COVID-19, the benefits of CCT in most clinical scenarios should not exceed the risks of exposure and infection of the personnel. Each case should be evaluated individually.

## 8. Nuclear Cardiology in the COVID-19 Era

Nuclear cardiology has a solid knowledge base on clinical experience, as well as diagnostic and prognostic values. All procedures in this area have the advantage of using widely automated protocols and equipment, which allows less contact between the health care professional and the patient. Moreover, except for exercise myocardial scintigraphy and ventilation/perfusion scintigraphy, no other nuclear medicine methods generate aerosols.^50^ These aspects could reduce exposure to the virus and infection propagation while also saving precious resources.^51^

### 8.1. Prioritization and Indications

Nuclear cardiology has a superior role in the pandemic scenario considering patients without COVID-19. The rationale for prioritizing examinations should be as described in Table 2. The main indications include:^11^

- Evaluating ischemia in patients with known CAD.
- Evaluating patients with chest pain syndromes. It is particularly useful for patients who are poor candidates for non-invasive anatomical imaging (such as those with stents or coronary artery calcification, who are allergic to contrast agents, or have a risk of impaired renal function).
- Evaluation of myocardial viability.
- Screening for amyloidosis.
- Identification of the inflammatory stages of sarcoidosis.
- Identification of infections in implanted devices.

On the other hand, a nuclear cardiology assessment is generally not necessary in the treatment of acute heart disease in patients with COVID-19. Ventilation scans or exercise stress tests should be omitted in areas going through the peak phase of the pandemic and/or in any patient with a known or suspected COVID-19 infection due to the high risk of aerosol emission.^52^ Specific considerations on the ventilation/perfusion scintigraphy protocol are described below.

### 8.2. Ventilation/Perfusion Scintigraphy

Currently, the gold standard for ruling out PE in patients with COVID-19 is the CT pulmonary angiography. However, this method cannot be used in patients with contraindications for iodinated contrast agents. A potential alternative relies in lung perfusion single-photon emission computed tomography (SPECT) using macroaggregated albumin tagged with ^99m^Tc. Due to the high risk of aerosol production associated to ventilation scintigraphy (aerosols tagged with ^99^mTc), the American Society of Nuclear Medicine and Molecular Imaging discouraged the classical combination of ventilation/perfusion images in patients with COVID-19.^53^ Ventilation scans should be omitted in patients with known or suspected COVID-19; therefore, the use of perfusion lung scintigraphy associated with chest CT or X-ray has been proposed.^54^

For optimizing protocols amidst the pandemic, one should follow good imaging practices that allow for a safe and efficient examination, as already described; these include mainly:

- Performing protocols that minimize the procedure duration without affecting test precision, eg, only using imaging stress tests when indicated.
- Avoiding protocols that may generate aerosols – give preference to pharmaceutical stress over exercise stress whenever possible.

Figure 5 is a proposition that summarizes the general protocol for reintroducing cardiovascular imaging examinations. Firstly, it is necessary to define whether the test is essential at any given moment. If it is essential (urgent or high-priority), define whether the patient has a high risk for COVID-19 and which method would lead to the lowest risk of exposure, even with the use of PPE. In case the examination is not essential/urgent, evaluate the local stage of the pandemic: when the area is at the peak stage, defer lower priority examinations. If the pandemic is decelerating in the area in question, reintroduce test scheduling according to the patient's risk for COVID-19 and indication priority, within appropriate criteria; finally, define PPE and patient flow according to COVID-19 risk and type of test. Tables 1, 2, and 3 summarize the indication prioritization, safety protocols, and PPE to be used according to the type of test and COVID-19 status. Among cardiovascular imaging examinations, echocardiography is considered first-line. However, in view of the need for minimizing the examining physician's exposure to SARS-COV-2 and rationally using available resources, alternative methods can be used to answer the clinical question, especially in stable patients or those with uncertain symptoms or who were referred to other imaging examinations.^8^

**Figure 5:** Flowchart for reintroducing cardiovascular imaging examinations in the COVID-19 era

## 9. Conclusion

The COVID-19 pandemic has forced the medical community to reconsider performing cardiovascular imaging examinations. Adaptations and changes were necessary due to the worldwide impact of this pandemic. The return to “normality” in cardiovascular imaging services should be progressive and adapted to regional differences within the country. Considering the impact of cardiovascular diseases in the population morbidity and mortality, cardiovascular signs and symptoms cannot be neglected. Therefore, even amid a pandemic situation where all attention is turned to fighting COVID-19, patients and physicians should be encouraged to perform cardiovascular investigations and be assured that these will happen in a safe environment. This position statement reflects the opinion of specialists based on national and international guidelines and the scientific evidence available at the moment, seeing that knowledge on COVID-19 is constantly evolving. In this environment, recommendations can guide the health care team and protect patients and professionals without compromising care. In addition, these recommendations, the constant dialog between medical imaging specialists, clinical staff, and patients constitutes the best and most efficient measure for facing the COVID-19 pandemic.
